# *Lactobacillus reuteri* suppresses *E*. *coli *O157:H7 in bovine ruminal fluid: Toward a pre-slaughter strategy to improve food safety?

**DOI:** 10.1371/journal.pone.0187229

**Published:** 2017-11-01

**Authors:** Yolande Bertin, Chloé Habouzit, Lysiane Dunière, Marie Laurier, Alexandra Durand, David Duchez, Audrey Segura, Delphine Thévenot-Sergentet, Federico Baruzzi, Frédérique Chaucheyras-Durand, Evelyne Forano

**Affiliations:** 1 Université Clermont Auvergne, INRA, MEDIS, Clermont-Ferrand, France; 2 Lallemand SAS, Blagnac, France; 3 Institut Pascal—Axe GePEB, Polytech Clermont-Ferrand, Université Blaise Pascal, Aubière, France; 4 Research Group on Bacterial Opportunistic Pathogens and Environment, UMR, Ecologie Microbienne, CNRS, VetAgro Sup, INRA and Université Lyon 1, Villeurbanne, France; 5 Laboratoire d'Étude des Microorganismes pathogènes, French Laboratory for Shiga Toxin-Producing Escherichia coli, VetAgro Sup, Campus vétérinaire, Marcy L’Etoile, France; 6 Institute of Sciences of Food Production, National Research Council of Italy, Bari, Italy; University of Connecticut, UNITED STATES

## Abstract

The bovine gastrointestinal tract (GIT) is the main reservoir for enterohaemorrhagic *Escherichia coli* (EHEC) responsible for food-borne infections. Therefore, it is crucial to develop strategies, such as EHEC suppression by antagonistic microorganisms, to reduce EHEC survival in the GIT of cattle and to limit shedding and food contamination. Most human-derived *Lactobacillus reuteri* strains produce hydroxypropionaldehyde (HPA), an antimicrobial compound, during anaerobic reduction of glycerol. The capacity of *L*. *reuteri* LB1-7, a strain isolated from raw bovine milk, to produce HPA and its antimicrobial activity against an O157:H7 EHEC strain (FCH6) were evaluated in bovine rumen fluid (RF) under strict anaerobiosis. EHEC was totally suppressed when incubated in RF inoculated with *L*. *reuteri* LB1-7 and supplemented with 80 mM glycerol (RF-Glyc80). The addition of LB1-7 or glycerol alone did not modify EHEC survival in RF. Glycerol was converted to HPA (up to 14 mM) by LB1-7 during incubation in RF-Glyc80, and HPA production appeared to be responsible for EHEC suppression. The bactericidal activity of *L*. *reuteri* LB1-7, the concentration of glycerol required and the level of HPA produced depended on physiological and ecological environments. *In vitro* experiments also showed that EHEC inoculated in rumen fluid and exposed to *L*. *reuteri* and glycerol had a very limited growth in rectal contents. However, *L*. *reuteri* exerted an antimicrobial activity against the rumen endogenous microbiota and perturbed feedstuff degradation in the presence of glycerol. The potential administration of *L*. *reuteri* and glycerol in view of application to finishing beef cattle at the time of slaughter is discussed. Further *in vivo* studies will be important to confirm the efficiency of *L*. *reuteri* and glycerol supplementation against EHEC shedding in ruminants.

## Introduction

Enterohaemorrhagic *Escherichia coli* (EHEC) are Shiga toxin-producing *E*. *coli* (STEC) responsible for severe human diseases such as haemolytic uraemic syndrome [1]. The majority of infections, commonly attributed to the consumption of contaminated food is caused by EHEC with serotype O157:H7 and the gut of ruminants, mainly cattle, is considered as the principal reservoir [2]. Therefore, it is important to develop nutritional or ecological strategies to reduce EHEC survival in the gastrointestinal tract (GIT) of cattle and to limit shedding and further contamination of food products.

Several approaches have been proposed to reduce the prevalence of *E*. *coli* O157:H7 in cattle, including feeding of antagonistic microorganisms, vaccination or bacteriophage treatment. Lactobacilli, which are known to exhibit an inhibitory effect against various enteric pathogens, are widely used as probiotics or direct-fed microbials in humans and animals [3, 4]. Lactobacilli display antimicrobial activities as a result of production of metabolites such as lactic acid, bacteriocins or other non-proteinaceous molecules [4].

*Lactobacillus reuteri* is used commercially as a probiotic microorganism and possesses antimicrobial properties against intestinal pathogens. It is well documented that specific *L*. *reuteri* can produce antimicrobial factors, such as hydroxypropionaldehyde (HPA) (also named reuterin), reutericyclin, reutericin, lactate or Mucus Adhesion-Promoting (MAP) protein [5, 6]. HPA has been proven effective against bacteria, fungi and protozoa survival [7, 8]. It has been postulated that HPA inhibits the activity of bacterial ribonucleotide reductase, a key enzyme catalysing the first step in DNA synthesis, which would explain the broad-spectrum activity of HPA [7, 9].

HPA is produced by *L*. *reuteri* during a two-step anaerobic fermentation of glycerol: a glycerol dehydratase first catalyses the conversion of glycerol to HPA and HPA is then reduced to 1,3 propanediol (1,3-PD) [7, 8]. In addition, *L*. *reuteri* is known to excrete HPA in high amounts when the level of fermentable carbohydrates is low [10]. In germfree mice, *L*. *reuteri* administration reduced both colonization and clinical signs due to EHEC infection and resulted in HPA production in the caecum [11].

In cattle, the terminal recto-anal junction has been referred as the major site of *E*. *coli* O157:H7 multiplication [12]. However, O157:H7 isolates are found throughout the GIT compartments (including the rumen) of experimentally infected calves and naturally shedding cattle [13, 14]. In addition, O157:H7 isolates in the rectum are clonally similar to isolates found in the rumen and fecal shedding has been shown to be positively associated with the presence of *E*. *coli* O157:H7 in the rumen [15]. All these observations demonstrate that the rumen is a reservoir for the contamination of hindgut compartments. Cattle regurgitate digesta during the rumination process and *E*. *coli* O157:H7 in the rumen may directly participate to EHEC dissemination in environment and to animal-to-animal transmission. In addition, quorum sensing molecules homoserine lactones (AHLs) promote EHEC colonization of the bovine gut [16]. Interestingly, AHLs are produced by the rumen microbiota but are not found in other GIT compartments highlighting the role of the rumen in the successful EHEC colonization of the bovine gut [16]. For all these reasons, among strategies for decreasing EHEC burden in cattle GIT, it can be proposed to focus on reducing EHEC survival in the rumen.

Because the rumen of cows is under strict anaerobiosis and contains a low level of easily fermentable carbohydrates, we speculated that *L*. *reuteri* could produce HPA at this site. In this study, we explored for the first time the potential growth inhibition exerted by *L*. *reuteri* towards EHEC in bovine rumen fluid. We tested several *L*. *reuteri* strains and demonstrated that *L*. *reuteri* LB1-7 isolated from raw bovine milk [17] produced HPA and could achieve a total suppression of EHEC in rumen fluid. *In vitro* experiments described in this study also showed that the exposure of EHEC to *L*. *reuteri* and glycerol in rumen fluid decreased drastically EHEC inoculation of lower bovine digestive segments.

## Materials and methods

### Bacterial strains and growth conditions

*L*. *reuteri* and EHEC strains used in this study are listed in S1 Table. The EHEC strain FCH6 (O157:H7, *eae*^+^, *stx1*^-^, *stx2*^+^) had been isolated from a case of HUS in 2004 due to the consumption of raw milk cheese. EHEC strains were routinely cultured in Luria Bertani (LB) broth (Biokar, Beauvais, France). *L*. *reuteri* strains were routinely cultured in De Man, Rogosa and Sharpe (MRS) broth (Biokar, Beauvais, France). Spontaneous rifampicin-resistant mutants of *L*. *reuteri* and EHEC strains were isolated on MRS agar (pH 6.8) and LB agar plates containing 100 μg/mL of rifampicin (Sigma-Aldrich, St Quentin Fallavier, France), respectively. Each wild-type strain and its corresponding spontaneous Rif^R^ mutant showed identical growth patterns. Sensitivity of the rumen microbiota to rifampicin was confirmed by spotting 100 μL of rumen fluid (RF) samples on MRS agar plate containing rifampicin (50 μg/mL) before incubation at 39°C for 24 hours. Survival of *L*. *reuteri* in RF was tested by incubating RF samples (from O_2_-free, CO_2_-saturated sterile flasks) inoculated with *L*. *reuteri* Rif^R^ under anaerobiosis (39°C). The next day, the RF samples were 10-fold serially diluted in sterile phosphate buffered saline (PBS) buffer (pH 7.4), plated on MRS agar plates containing rifampicin and incubated overnight under anaerobiosis at 37°C for CFU counting. The experiments were replicated three times.

### Ethics statement

RF and Rec samples were collected from rumen-cannulated cows in the INRA “Herbipôle Experimental Unit”, Auvergne-Rhône-Alpes Research Centre, Saint Genès-Champanelle, France (agreement number: C6334517) for experiments specifically approved by the “Comité d’éthique en matière d’expérimentation animale en Auvergne” (C2EA-02, permit number: *6895-*2016091913586944V3). The animals were housed in individual stalls in the INRA “Herbipôle” experimental facilities in accordance with the guidelines of the local ethics committee. Small intestine and caecum contents were collected after the slaughter of animals required in the experimental slaughterhouse of the INRA “Herbipôle Experimental Unit” (permit number: APAFIS#1765–2015091516305 v3). Animals were slaughtered in accordance with the guidelines of the local Ethics Committee and current INRA ethical guidelines for animal welfare by stunning immediately followed by jugular vein bleeding performed by specifically trained abattoir staff (Permit number: 63345001).

### Animals and digestive contents sampling

RF samples and rectum contents (Rec) were collected from non-lactating rumen-cannulated Holstein cows (live weight 800 kg on average). They were fed a diet composed of meadow hay and pelleted concentrate (INRA 50183 GV) and 250 g of mineral and vitamin complex. Cows were fed 8 kg of hay, 2 kg of concentrate (DM) in one meal, distributed at 8:00 in the morning.

RF samples were collected manually before feeding through the rumen cannula in O_2_-free, CO_2_-saturated sterile and pre-warmed (39°C) flasks as previously described [18]. The samples were immediately filtered through four layers of cheesecloth into CO_2_-saturated sterile flasks. RF samples (pH 6.2 on average) were confirmed to be negative for O157 *E*. *coli* before experiments: i) no colony was obtained after 24 h of incubation of the samples on MacConkey agar containing sorbitol (SMAC) and ii) the genes *stx2*, *rfbE* and *fliC* were not amplified from RF DNA (oligonucleotides used in PCR amplification were described in S2 Table). Bacterial counts from freshly collected RF samples were performed under anaerobiosis using the Most Probable Number (MPN) method and revealed the presence of ≈ 5 x 10^10^ MPN/mL. At least two cows at three different days were used for each experiment with RF samples. After sampling, rectum contents were rapidly transferred to O_2_-free, CO_2_-saturated sterile flasks, diluted (1:1) in sterile reduced potassium phosphate buffer (50 mM potassium phosphate, resazurin 0.1%, 40 mM Na_2_CO_3_, 3 mM cysteine, pH 7) and used immediately. Three different cows were used for experiments with Rec samples. Small intestine and caecum contents were collected from three cows at slaughter as previously described [19], rapidly transported to the laboratory, pooled in equal proportions and stored at -20°C until use.

### Agar spot test

An agar spot test was performed to detect antimicrobial activity of *L*. *reuteri* strains as previously described [20]. The antimicrobial activity was recorded as growth-free inhibition zones (diameter > 1 mm) around the spots.

### *In vitro* HPA production by *L*. *reuteri*

HPA was produced under anaerobiosis in PBS buffer (pH 7.4) and RF samples using a two-step fermentation protocol [20]. Briefly, RF samples were first centrifuged twice at 4100 *g* for 20 min and the supernatants were filter-sterilized through 0.45-μm nylon filter (Merck-Millipore, St Quentin en Yvelines). The filtrate was placed into glass tubes and left without stoppers in an anaerobic chamber (Jacomex, France) (100% CO_2_) during three days to allow the rumen fluid filtrate to be under anaerobic conditions. The tubes were then recapped with butyl rubber stoppers and filtered again (0.45 μm) under CO_2_ flux. *L*. *reuteri* strains were incubated in MRS broth without shaking under anaerobiosis for 24 hours at 37°C. The cultures were then centrifuged (4100 *g*, 10 min), washed in sterile PBS buffer and adjusted to ≈ 10^9^ CFU/mL in O_2_-free, CO_2_-saturated PBS or filter-sterilized RF samples supplemented with 250 mM glycerol (Fisher-Aldrich). After incubation for 2 hours at 37°C, the bacterial cultures were pelleted and HPA-containing supernatants were collected and filtered (0.22-μm) before storage at 4°C.

### HPA quantification

HPA production was quantified by a tryptophan-HCl colorimetric assay as previously described [10]. Briefly, the HPA-containing supernatants were diluted (10-fold) in sterile H_2_O, mixed with 10 mM tryptophan solution (0.01 M in 0.05 HCl, stabilized with a few drops of toluene) and 12 M HCl before incubation for 30 min at 37°C. Optical density (OD_560_) was measured with a spectrophotometer Spectronic BioMate^TM^ 3 (Thermo Scientific). Acrolein (Fisher-Aldrich) was used as standard (the method is specific to both reuterin and acrolein quantification [10]). Standard curves were generated using an acrolein solution diluted to a concentration range of 0–20 mM in PBS or filter-sterilized RF. All the samples were filtered (0.45 μm) and diluted in PBS buffer if necessary.

### Co-incubation of EHEC and *L*. *reuteri* strains

Pre-cultures of EHEC FCH6 Rif^R^ or EDL933 Rif^R^ inoculated from a single colony were incubated aerobically overnight at 37°C in LB supplemented with rifampicin. Bacterial cultures were then centrifuged (4100 *g*, 10 min) and the pellets were resuspended in sterile PBS buffer. At the same time, *L*. *reuteri* strains were cultured in MRS broth for 24 hours (37°C) without shaking. RF samples or LB broth (5 mL) were introduced into glass tubes equipped with butyl stoppers and screwed caps (Hungate tubes) under a 100% CO_2_ atmosphere. Ground feed (25 mg) was then added to RF samples (80% meadow hay, 20% concentrate) to mimic a feeding cycle. RF samples or LB broth were inoculated with both *L*. *reuteri* (≈ 10^7^ CFU/ml) and the EHEC Rif^R^ (≈ 10^4^ CFU/mL) strains. To mimic the physiological conditions encountered in the rumen, the cultures were incubated under strict anaerobiosis at 39°C (bovine temperature) for 24 hours (≈ transit time of forage-rich digesta) with gentle shaking (mixing of digesta). The bacterial cultures were then 10-fold serially diluted in PBS and spotted (10 μL) in triplicate on LB plates containing rifampicin before incubation overnight at 37°C. Each experiment was performed independently at least three times. The values presented are the log_10_ mean number of CFU/mL.

### *In vitro* feedstuff degradation by the rumen microbiota

The effect of *L*. *reuteri* on feedstuff degradation by the rumen microbiota was assessed using the *in vitro* Daisy II incubation system (ANKOM Technology Corporation, Fairport, NY, USA). This system allows evaluation of feed digestibility during simultaneous incubation of up to 4 glass vessels, which rotate in an insulated chamber maintained at 39.5°C (≈ rumen temperature) [21–23]. RF samples were diluted (1:4, vol:vol) with Goering and Van Soest (GVS) anaerobic buffer [24] and dispensed under O_2_-free CO_2_ atmosphere. Feedstuff, under the form of alfalfa hay (AH) or corn silage (CS) (0.25 g of each forage, ground to 1 mm particle size), was bagged (ANKOM filter bags F57, porosity 25 μm, 5.0 cm×5.5 cm) and incubated in the presence of diluted RF for 24 hours. Six glass beads (4 mm diameter) were added to each bag to improve bag immersion in the rumen fluid as previously described [22]. All filter bags were heat-sealed at 0.5 mm from the edge of the bags. One bag with only 6 glass beads was also placed in each jar as blank. Six replicates for each feedstuff were used, and the experiment was repeated three times with RF collected at one-week intervals. For each replicate, one vessel was used as control and contained only RF, buffer, AH and CS bags, and one was inoculated with ≈ 10^7^ CFU/mL of *L*. *reuteri* LB1-7 and 80 mM glycerol at the start of incubation. *L*. *reuteri* suspension was prepared from an overnight MRS culture at 37°C. The culture was centrifuged and resuspended in GVS buffer. pH was monitored at the start and end of incubation in a 10 mL sample of the incubation mix. Samples were collected and stored (-20°C) for fermentation end products analysis and microbial populations quantification by qPCR (see sections below). After 24 hours of incubation, bags were removed, washed with tap water and dried at 65°C for at least 48 hours for determination of residual DM. Six bags of each feedstuff were not incubated in order to assess passive DM loss by tap water washing. It was then possible to calculate the disappearance of DM (%).

### Analysis of fermentation products in the RF samples

Metabolites were quantified by high performance liquid chromatography (HPLC) as previously described [25]. The method was calibrated to detect acetate, citrate, pyruvate, fructose, glucose, lactate, glycerol, 1,3-PD, propionate, isobutyrate, ethanol, butyrate, isovalerate and valerate. The amount of undissociated lactate was calculated using the formula: undissociated acid (mM) = total acid (mM) / (1+10^pH-pka^) [26]. The pH value of RF was 6.2 and the pKa value of lactate is 3.86.

### Incubation of EHEC in rectum contents

Survival of EHEC during its passage in different segments of the bovine GIT was simulated. To this, we performed a first incubation of EHEC in filter-sterilized RF samples (FS-RF) (0.22 μM) followed by a second incubation in Rec samples (RF samples were indeed filtered in order to retrieve only the inoculated bacteria and not the feed particles neither the rumen endogenous microbiota). Briefly, RF samples were centrifuged, filter-sterilized and placed into glass tubes in an anaerobic chamber as described above. The strain FCH6 Rif^R^ (≈ 10^4^ CFU/mL) was incubated under strict anaerobiosis with gentle shaking in FS-RF samples (5 mL) inoculated or not with *L*. *reuteri* (≈ 10^7^ CFU/mL) and supplemented with 80 mM glycerol. After 24 hours of incubation at 39°C, the samples were centrifuged (4100 *g*, 10 min) and the bacterial pellet (containing potentially *L*. *reuteri* and/or EHEC) was suspended in sterile CO_2_-satured PBS (0.5 mL). The bacterial suspension was then inoculated into Rec samples freshly collected (containing its endogenous microbiota) (5 mL) and incubated at 39°C under anaerobiosis. The concentration of EHEC Rif^R^ was measured after 24h of incubation in FS-RF and after 6 and 24 hours of incubation in Rec samples.

### DNA extraction

Genomic DNA from pure culture of *L*. *reuteri* was extracted using the Easy-DNA^TM^ kit (Fisher Scientific, Illkirch, France). The *L*. *reuteri* strains were cultured aerobically for 24 hours in MRS broth at 37°C without shaking. The next day, the bacterial cultures were centrifuged (10,000 *g*, 15 min) and the pellet was washed twice in sodium phosphate buffer (50 mM, pH 8). Initial cell lysis was performed using proteinase (Easy-DNA^TM^ kit) for 5 min at 37°C. Bacterial cells were then disrupted by bead beating for 3 min with 0.2 g of zirconia beads (0.1 mm diameter, Sigma-Aldrich). Subsequent genomic DNA extraction was performed according to the manufacturer’s recommendations.

Total DNA from the rumen and intestinal contents was extracted as previously described [27]. Briefly, bacterial cells were suspended in buffer (50 mM Tris buffer, 0.5 M NaCl, 50 mM EDTA, 4% SDS) (pH 8) and disrupted by bead beating for 3 min with zirconia beads. The mixture was incubated at 70°C for 15 min and centrifuged (16,000 *g* for 2 min at 15°C). The resulting supernatant was then precipitated, washed and resuspended as previously described [27]. Contaminating RNA was removed using a DNAse-free RNAse (10 mg/mL) and an additional incubation for 15 min at 37°C with 15 μL of Proteinase K (20 mg/mL, Sigma-Aldrich) was also performed. Subsequent DNA purification was performed using the Qiamp Fast DNA Stool mini kit (Qiagen, Courtaboeuf, France).

### Oligonucleotide primer design and PCR screening

*L*. *reuteri* genes were detected by PCR amplification using the primer pairs described in S2 Table. The primers used to detect *Lactobacillus* spp. target a 16S rRNA sequence conserved among *Lactobacillus* spp. but also *Leuconostoc* spp., *Pediococcus* spp. and *Weissella* spp. [28]. Therefore, in this study, the bacteria detected by these primers were designed as *Lactobacillus* group. DNA sequences from *L*. *reuteri* available in databases (JCM 1112 [AP007282.1], DSM 20016 [CP000705.1], FUA3400 [KJ435307.1], SD2112 [CP002844.1], BR11 [GU191838.1] and BPL36 [JQ897939.1]) were aligned (Align Sequences Nucleotide BLAST [http://blast.ncbi.nlm.nih.gov]) to design the primer pairs used to amplify the genes *gldC* and *dhaT* (S2 Table). Amplification was performed from genomic DNA extracted from *L*. *reuteri* strains as described above. Taq polymerase and enzyme buffer were from MP-Biomedicals (Illkirch, France). The PCR procedure was performed on a Mastercycler Personal apparatus (Eppendorf) using the following programme: initial denaturation at 94°C for 4 min, 30 cycles of 94°C for 30 s, 55°C for 30 s, and 72°C for 1 min, and a final elongation at 72°C for 10 min.

### Bacterial enumeration in RF samples by quantitative-PCR

Total bacteria, *Fibrobacter succinogenes*, *Ruminococus flavefaciens*, *L*. *reuteri* and the *Lactobacillus* group were quantified by quantitative PCR (q-PCR). The q-PCR quantification was performed as previously described [27]. The primer pairs targeting the *rrs* and *hsp60* genes are described in S2 Table. The standard curves (10^8^ to 10^3^ *rrs* or *hsp60* copies) targeting *Lactobacillus* group and *L*. *reuteri*, respectively, were established from genomic DNA extracted from *L*. *plantarum* and *L*. *reuteri* strains. DNA extracted from RF samples inoculated with LB1-7 and *L*. *plantarum* (≈ 10^6^ CFU/mL) was used as positive and negative control respectively for *L*. *reuteri* quantification. The standard curves (10^8^ to 10^3^ *rrs* copies) targeting *R*. *flavefaciens* and *F*. *succinogenes* and total bacteria were established as previously described [27].

### Statistical analysis

Values are expressed as mean ± SEM. Statistical analyses were performed with the GraphPad Instat statistical software (La Jolla, CA, USA). Student's *t*-test was used to compare means and discuss the effect of *L*. *reuteri*. All tests were two-tailed paired and the level used to establish significance was *P* < 0.05.

## Results

### Survival of EHEC in bovine rumen fluid

The fate of the spontaneous rifampicin mutants of EHEC FCH6 and EDL933 (S1 Table) was assessed in rumen fluid (RF) containing endogenous microbiota under *in vitro* culture conditions allowing the growth of the endogenous microbiota and mimicking the rumen physiological conditions (see the Materials and Methods section). A concentration of ≈ 10^4^ EHEC/mL was chosen because 3–6 log_10_ colony-forming unit (CFU)/mL were enumerated from the rumen of steers experimentally infected with *E*. *coli* O157:H7 [29]. Results analysis showed that FCH6 Rif^R^ survived well after incubation in RF samples (only 0.7 log_10_ decrease in CFU/mL after 24 hours of incubation) whereas the spontaneous Rif^R^ mutant of the reference EHEC strain EDL933 (EDL933 Rif^R^) showed higher cell death after anaerobic incubation in RF (1.9 log_10_ decrease in CFU/mL) (S1 Fig). The strain FCH6 was thus selected for further studies.

### Ability of *L*. *reuteri* to produce HPA and to inhibit EHEC growth

Five *L*. *reuteri* strains (S1 Table) were tested for their ability to inhibit the growth of EHEC FCH6 on soft BHI agar plates. Results showed that FCH6 was susceptible to *L*. *reuteri* strains LB1-7 and F275 under anaerobiosis when 2% glycerol (217 mM) was added whereas it was poorly or not inhibited by the remaining *L*. *reuteri* strains, with or without glycerol supplementation (S2 Fig). *L*. *reuteri* strains were also tested by PCR to detect the genes *gldC* and *dhaT* encoding glycerol dehydratase large subunit (EC 4.2.130) (required to convert glycerol to HPA) and 1,3-propanediol dehydrogenase (EC 1.1.1.202) (required to reduce HPA to 1,3-PD), respectively. Amplified products with the expected size for *dhaT* were obtained from all the *L*. *reuteri* strains tested, whereas the amplicon specific for *gldC* was only obtained for *L*. *reuteri* LB1-7, F275 and 65A (S3 Fig). In accordance with the results of agar spot assay, *gldC* was not amplified from *L*. *reuteri* F70 and 100–23 genomic DNA. The capacity of *L*. *reuteri* strains to produce HPA was then tested in PBS buffer supplemented with 250 mM glycerol. Results showed that HPA production at 37°C under anaerobiosis was strain dependent since LB1-7, F275 and 65A produced 114, 84 and 19 mM HPA, respectively. As expected, HPA was not detected in the supernatant of *L*. *reuteri* strains 100–23 and F70. The production of HPA by *L*. *reuteri* was also tested in filter-sterilized RF samples supplemented with 250 mM glycerol: the strains LB1-7 and F275 produced 189 and 105 mM HPA, respectively, whereas HPA was not detected in the presence of *L*. *reuteri* strains 65A, 100–23 and F70. In view of these results, the *L*. *reuteri* strain LB1-7 was chosen to perform co-incubation experiments. The *L*. *reuteri* strain 100–23 was used as negative control.

### Persistence of *L*. *reuteri* in bovine intestinal fluids

The quantification of *L*. *reuteri* and *Lactobacillus* group in intestinal contents was performed by qPCR amplification of the genes *hsp60* (encoding heat shock protein) and *rrs* (encoding 16S ribosomal RNA), respectively. *Lactobacillus* group was detected in RF samples (7.5 log_10_ *rrs* copy per μg of DNA ± 0.16), small intestine contents (7.8 log_10_ *rrs* copy per μg of DNA ± 0.3), caecum contents (7.4 log_10_ *rrs* copy per μg of DNA ± 0.09) and rectum contents (7.6 log_10_ *rrs* copy per μg of DNA ± 0.05). In contrast, *L*. *reuteri* was undetectable in all GIT compartments tested, including the rumen (detection limit: 2 log_10_ *hsp60* copies). The capacity of *L*. *reuteri* LB1-7 to grow in RF samples was then analyzed. After 24 hours of incubation in RF at 39°C under strict anaerobiosis, the spontaneous rifampicin-resistant mutants of LB1-7 (LB1-7 Rif^R^) was enumerated at a concentration similar to the inoculation rate (≈ 10^7^ CFU/mL), demonstrating the capacity of the strain to persist in RF.

### Antimicrobial activity of *L*. *reuteri* and antimicrobial potency of HPA against EHEC

**Fig 1:**
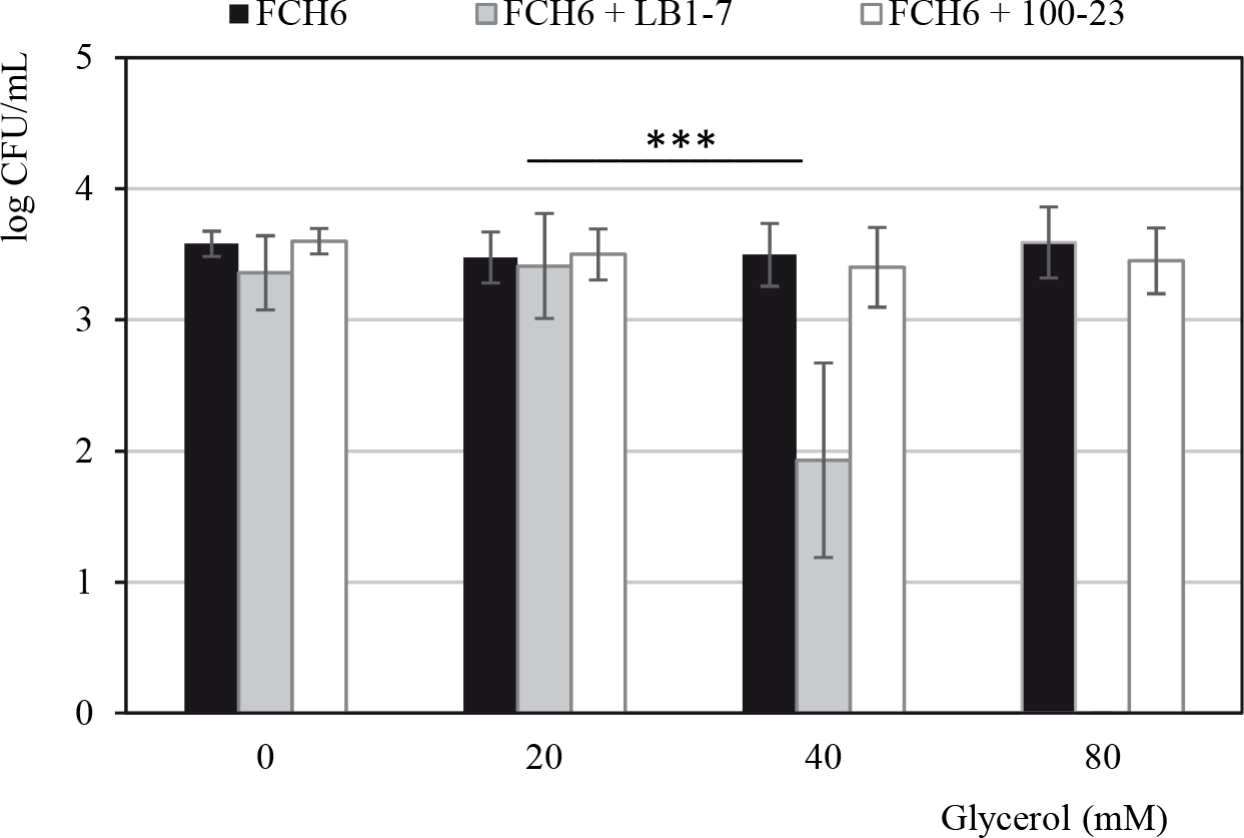
Survival of EHEC co-incubated with *L*. *reuteri* strains in RF samples supplemented or not with glycerol. The strain FCH6 Rif^R^ was co-inoculated with ≈ 10^7^ CFU/mL of *L*. *reuteri* LB1-7 (HPA producer) or 100–23 (negative control) in RF samples under anaerobiosis for 24 hours. The strain FCH6 Rif^R^ inoculated alone in RF samples was used as control. RF samples were supplemented or not with glycerol at different concentrations. Bars represent the SEM of three independent experiments. Asterisks indicate statistical significance (***: P<0.001).

The growth of EHEC FCH6 Rif^R^ (≈ 10^4^ CFU/mL) was tested in the presence of different concentrations of *L*. *reuteri* LB1-7 (≈ 10^5^, 10^6^ and 10^7^ CFU/mL) and glycerol (20, 40, 80 and 160 mM) in RF samples to determine the optimal conditions to decrease EHEC survival. The strain FCH6 Rif^R^ survived well after 24 hours of anaerobic incubation in RF samples in the absence of glycerol or supplemented with 20 or 40 mM glycerol whatever the *L*. *reuteri* concentration (S3 Table). In contrast, no EHEC CFU was enumerated when LB1-7 (10^7^ CFU/mL) was inoculated in RF supplemented with 80 or 160 mM glycerol (detection limit: 10 CFU/mL) (Fig 1). The inhibition of higher levels of EHEC (≈ 10^5^ and 10^6^ CFU/mL) were also tested. *L*. *reuteri* LB1-7 (≈ 10^7^ CFU/mL) suppressed EHEC FCH6 in RF supplemented with 80 mM glycerol (RF-Glyc80) whatever the EHEC concentration. Similarly, co-incubation of *L*. *reuteri* LB1-7 (≈ 10^7^ CFU/mL) and EDL933 Rif^R^ (≈ 10^4^ CFU/mL) in RF-Glyc80 also resulted in EHEC suppression after 24 hours of anaerobic incubation. As expected, *L*. *reuteri* 100–23 (≈ 10^7^ CFU/mL) had no effect on EHEC survival with or without glycerol (Fig 1). Results also showed that glycerol alone was not responsible for EHEC suppression since *L*. *reuteri* LB1-7 Rif^R^ showed similar growth curves when incubated in RF and RF-Glyc80. This demonstrated the capacity of *L*. *reuteri* to persist in RF in the presence of glycerol. Different dilutions of HPA-containing supernatants were added to RF samples inoculated with FCH6 Rif^R^ to test the antimicrobial potency of exogenous HPA. Addition of 25 mM HPA in RF samples (not inoculated with LB1-7) completely suppressed FCH6 Rif^R^ after 2 hours of incubation.

### Production of HPA by *L*. *reuteri* in rumen fluids

**Fig 2:**
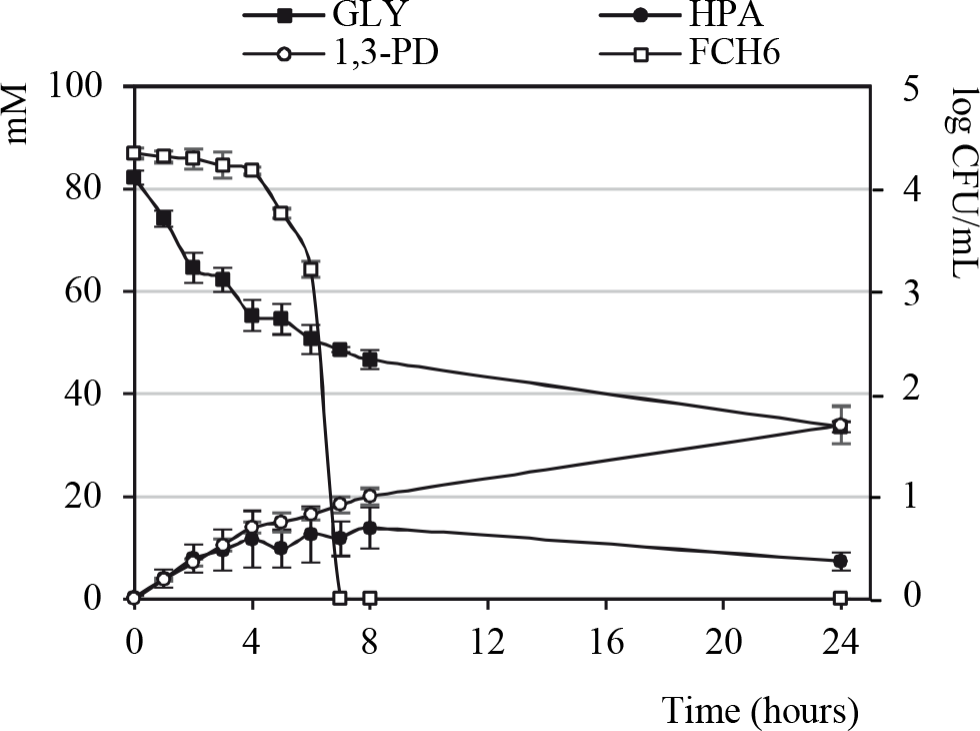
EHEC counts and HPA, 1,3-PD and glycerol quantification in RF-Glyc80. The strain FCH6 Rif^R^ (≈ 10^4^ CFU/mL) was co-incubated with *L*. *reuteri* LB1-7 (≈ 10^7^ CFU/mL) in RF samples supplemented with 80 mM glycerol under anaerobiosis. At each time point the strain FCH6 Rif^R^ was enumerated and accumulation of HPA and 1,3-PD, and disappearance of glycerol were monitored. Bars represent the SEM of three independent experiments. Gly: glycerol.

Kinetics of EHEC disappearance and HPA accumulation were monitored during co-incubation of FCH6 Rif^R^ (≈ 10^4^ CFU/mL) and LB1-7 (≈ 10^7^ CFU/mL) in RF-Glyc80. As shown in Fig 2, a significant decrease in EHEC population was observed from 4 hours of co-incubation (P<0.05) and no EHEC colony could be detected after 6 hours of co-incubation. A total suppression of EHEC was observed when HPA concentration reached ≈ 12.5 mM and a maximal HPA concentration (13.8 mM) was quantified after 8 hours of incubation (Fig 2). As expected, HPA and 1,3-PD accumulation correlated with glycerol disappearance and the glycerol concentration was only 51 mM and 34 mM in RF-Glyc80 after 6 and 24 hours of co-incubation, respectively. The concentrations of HPA and 1,3-PD accumulated in RF-Glyc80 were similar during the first 8 hours of incubation. Noteworthy, the level of 1,3-PD reached 34 mM after 24 hours whereas a decrease in HPA concentration was observed (7.3 mM) (Fig 2 and Table 1). These results suggested that HPA accumulation was transient probably because a part of HPA was reduced to 1,3-PD. As expected, HPA was not detected in the supernatant of RF-Glyc80 inoculated with *L*. *reuteri* 100–23 as well as in RF-Glyc80 samples not inoculated with *L*. *reuteri*.

Because Schaefer *et al*. show that HPA production by *L*. *reuteri* is stimulated by interaction with different bacteria [8], we investigated the potential stimulating role of EHEC in HPA production. A similar maximal concentration of HPA (≈ 14 mM HPA) was quantified at 8 hours of incubation of RF-Glyc80 co-inoculated with LB1-7 and FCH6 Rif^R^ or inoculated with LB1-7 alone demonstrating that the presence of EHEC in RF samples did not stimulate the production of HPA by *L*. *reuteri* under our experimental conditions.

**Table 1:** Concentration of glycerol and microbial metabolites.

	Acetate	Propionate	Butyrate		Glycerol		HPA	1,3-PD	Lactate
	(mM)^a^	(mM)^a^	(mM)^a^		(mM)^b^		(mM)	(mM)	(mM)
RF t = 0	61.2 ± 5.5	14.1 ± 1.6	6.3 ± 0.7		-		-	-	-
RF t = 24h	105.3 ± 2.9	33.7 ± 0.8	19.0 ± 1.1		-		-	-	-
RF + LB1-7 t = 24h	95.0 ± 2.4^NS^	29.9 ±0.9*	17.5 ± 1.8^NS^		-		-	-	1.8 ± 0.09
RF-Glyc80 t = 0	61.2 ± 5.5	14.1 ± 1.6	6.3 ± 0.7		82.0 ± 3.5		-	-	-
RF-Glyc80 t = 24h	95.8 ± 4.1^NS^	40.8 ± 1.6*	26.1 ± 1.7*		61.9 ± 5.8		-	2.6 ± 0.9	0.9 ± 0.35
RF-Glyc80 + LB1-7 t = 24h	79.6 ± 4.8*	17.8 ± 1.1***	7.8 ± 1.2**		34.2 ± 2.1*		7.3 ± 1.7	34.3 ± 2.8	28.7 ± 5.7
RF-Glyc80 + 100–23 t = 24h	ND	ND	ND		46.1 ± 1.9^NS^		-	3.5 ± 0.5	ND

Concentrations of major ruminal fermentation ends products, glycerol, HPA and 1,3-PD were quantified in RF and RF-Glyc80. The samples were inoculated or not with *L*. *reuteri* LB1-7. The detection limit of glycerol, 1,3-PD, lactate and HPA were 10^2^ mM, 2 x 10^2^ mM,10^2^ and 5 x 10^−2^ mM, respectively. Means of three independent replicates are shown with their SEM. ND: not determined; -: undetected.

^a^: comparisons were made with RF t = 24h

^b^: comparisons were made with RF-Glyc80 t = 24h

^NS^: P>0.05 (not significant)

*: P<0.05

**: P<0.01

***: P<0.001.

Values with no superscript were not statistically analyzed.

### Production of HPA and antimicrobial activity of *L*. *reuteri* LB1-7 in LB broth

**Fig 3:**
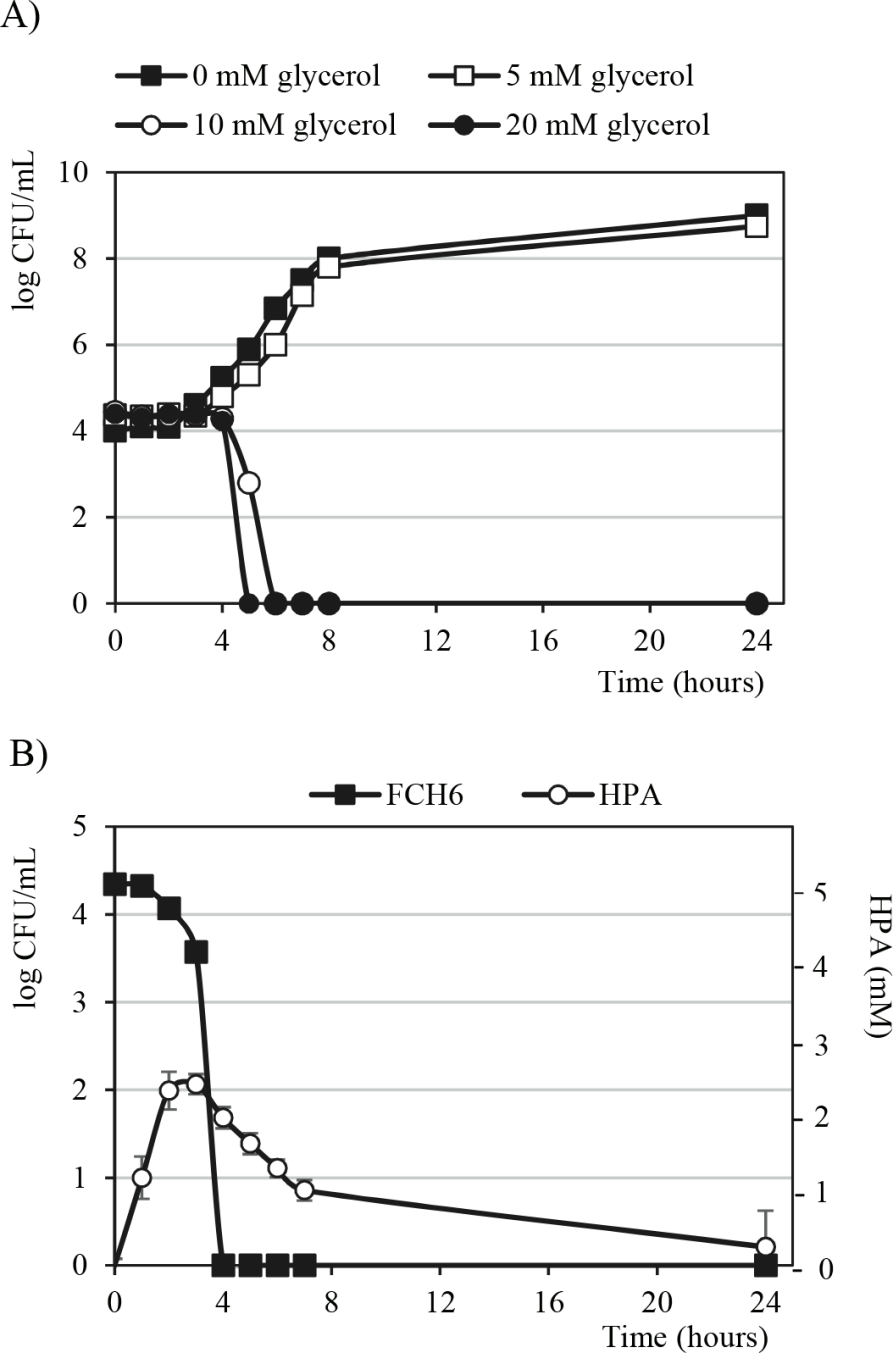
Kinetics of EHEC growth or disappearance and HPA production in LB broth. (A) The strain FCH6 Rif^R^ (≈ 10^4^ CFU/mL) was co-incubated with *L*. *reuteri* LB1-7 (≈ 10^7^ CFU/mL) in LB broth supplemented or not with different concentration of glycerol. The strain FCH6 Rif^R^ was then enumerated after 24 hours of incubation under anaerobiosis. Bacterial growth curves are expressed as a single representation of three independent experiments. (B) The strain FCH6 Rif^R^ was co-incubated with *L*. *reuteri* in LB broth supplemented with 10 mM glycerol under anaerobiosis. At each time point the strain FCH6 Rif^R^ was enumerated and accumulation of HPA was quantified. The bacterial growth curve is expressed as a single representation of three independent experiments. Bars represent the SEM of three independent experiments.

HPA production and antimicrobial activity of *L*. *reuteri* LB1-7 against EHEC was also investigated during co-incubation in sterile laboratory medium. As shown in Fig 3A, 10 mM glycerol was required to completely suppress FCH6 Rif^R^ co-inoculated with LB1-7 in LB broth after 6 hours of co-incubation. Up to 2.1 mM HPA was produced by *L*. *reuteri* LB1-7 after 3 hours of co-incubation with FCH6 Rif^R^ in LB broth supplemented with 10 mM glycerol (Fig 3B).

Taken together, the results showed that only 10 mM glycerol were required to completely suppress FCH6 Rif^R^ in LB medium inoculated with *L*. *reuteri* LB1-7 while 80 mM glycerol were necessary in RF. Furthermore, it is noteworthy that the HPA concentration produced by *L*. *reuteri* and apparently sufficient to suppress EHEC was 2.1 and 12.5 mM in LB broth and RF samples, respectively.

### Glycerol metabolism by endogenous rumen microbiota

Fermentation end products were analysed in RF samples. As expected, the three major short-chain fatty acids present in RF samples freshly collected were acetate, propionate and butyrate (RF t = 0, Table 1). The concentration of additional metabolites was below the detection limit: glucose (< 4 x 10^−3^ mM), glycerol (< 10^−2^ mM), 1,3-PD (< 2 x 10^−2^ mM), isobutyrate (< 10^−2^ mM), valerate (< 10^−2^ mM), lactate (< 10^−2^ mM) and ethanol (< 4 x 10^−2^ mM). Acetate, propionate and butyrate concentration increased after 24 hours of anaerobic incubation due to fermentation by the endogenous rumen microbiota of simple and complex carbohydrates provided by the ground feed added to RF samples (see the Materials and Methods section) (RF t = 24h, Table 1). The glycerol concentration was still ≈ 62 mM in RF-Glyc80 after 24 hours of incubation, indicating that the rumen microbiota assimilated ≈ 24% of the glycerol. In addition, incubation of RF-Glyc80 resulted in an altered fermentation pattern when compared with RF samples not supplemented with glycerol: disappearance of glycerol was associated with an increase in propionate and butyrate concentration (P<0.05) (Table 1). The 1,3-PD concentration was 2.6 mM in RF-Glyc80 after anaerobic incubation suggesting that the rumen microbiota was able to reduce glycerol to 1,3-PD to some extent. The HPA concentration in RF samples was below the detection limit (< 5 x 10^−2^ mM).

### Impact of *L*. *reuteri* on rumen fermentation and glycerol metabolism

The fermentation pattern of RF-Glyc80 samples inoculated with *L*. *reuteri* was analyzed (Table 1). After 24 hours of incubation under anaerobiosis, the glycerol concentration was 34.2 mM in RF-Glyc80 inoculated with LB1-7 (Table 1). Comparison with the level of glycerol recovered after incubation of non-inoculated RF-Glyc80 (61.9 mM) suggested that LB1-7 was able to metabolize glycerol, while the change in glycerol concentration in the presence of *L*. *reuteri* 100–23 was not significant. As expected, HPA was detected in RF-Glyc80 inoculated with LB1-7 but not with 100–23 (Table 1). Furthermore, as shown above, a relatively high concentration of 1,3-PD (34.3 mM) was quantified in RF-Glyc80 inoculated with *L*. *reuteri* LB1-7 whereas the level of 1,3-PD quantified in RF-Glyc80 inoculated with *L*. *reuteri* 100–23 was close to that obtained after incubation of RF-Glyc80 alone (Table 1). This suggests that the slight 1,3-PD accumulation observed in RF-Glyc80 inoculated with 100–23 was essentially due to anaerobic reduction of glycerol by the endogenous rumen microbiota. Similar patterns were obtained when RF-Glyc80 was inoculated with *L*. *reuteri* alone or co-inoculated with both *L*. *reuteri* and EHEC FCH6 Rif^R^.

In the absence of glycerol, incubation of RF with or without *L*. *reuteri* led to similar concentrations of acetate and butyrate, and a lower concentration of propionate (P<0.05) (Table 1). In contrast, incubation of *L*. *reuteri* LB1-7 in RF-Glyc80 led to lower concentrations of acetate (P<0.05), butyrate (P<0.01), and propionate (P<0.001) (Table 1). Noteworthy, lactate accumulation (28.7 mM; 0.13 mM undisociated from) was observed when LB1-7 was inoculated in RF-Glyc80 but not in RF.

To analyze the role of lactate in EHEC inhibition, the strain FCH6 Rif^R^ was incubated in RF-Glyc80 samples supplemented with 30 mM lactate. After 24 hours of incubation under anaerobiosis, a similar EHEC quantification (≈ 10^4^ CFU/mL) was recovered in RF samples supplemented or not with 30 mM lactate suggesting that the LB1-7 bactericidal action was not due to lactate production.

### Impact of *L*. *reuteri* on the degrading activity of the endogenous rumen microbiota

**Fig 4:**
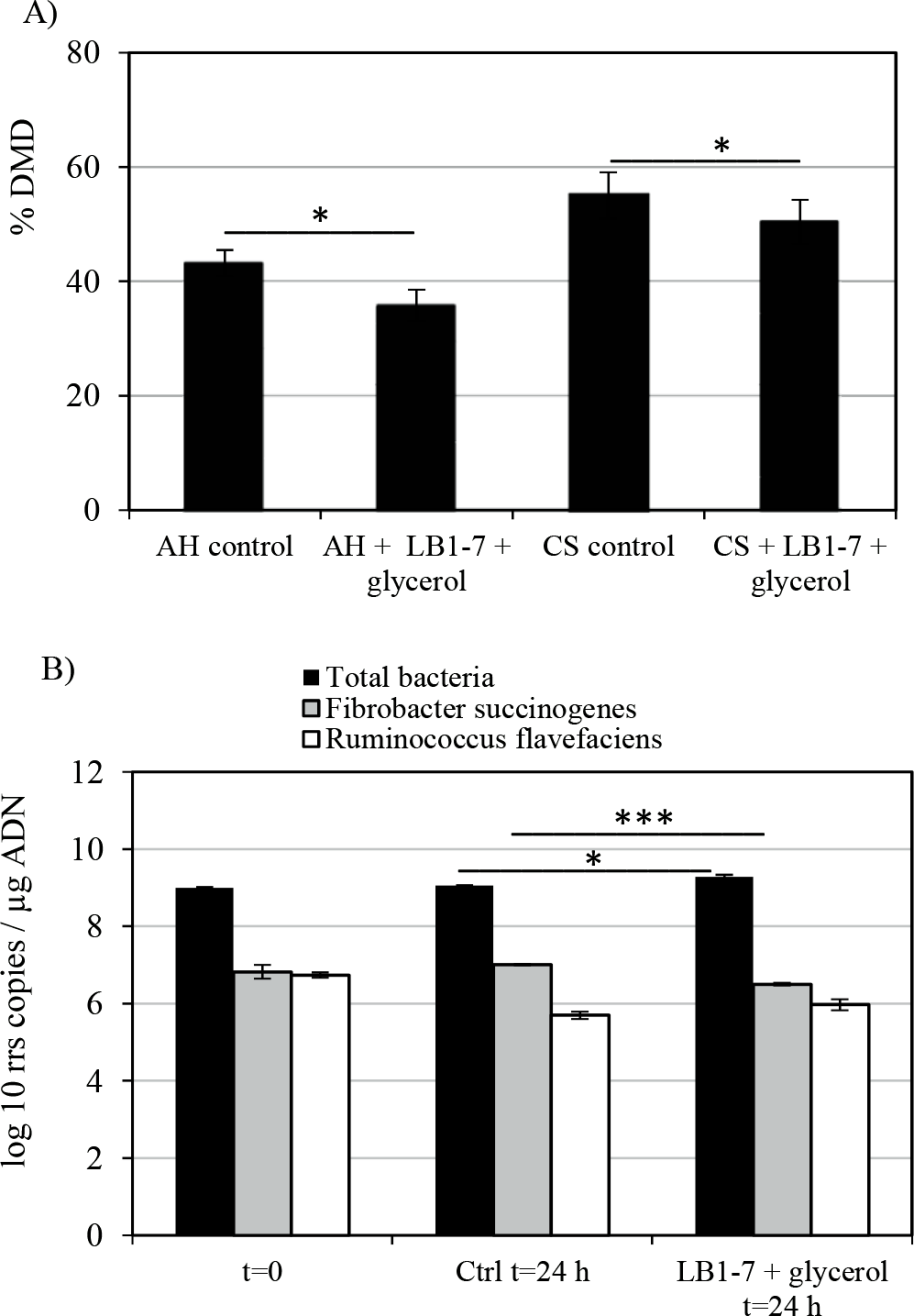
Dry matter degradation (DMD) of forages and fibrolytic ruminal population. (A) Dry matter degradation of alfalfa hay (AH) and corn silage (CS) by the rumen microbiota after 24 hours of incubation was quantified in the Daisy II incubation system containing RF in the presence or absence of *L*. *reuteri* LB1-7 and 80 mM glycerol. Non-incubated bags containing forage were used as control (see the experimental procedures section). The data are expressed as the percentage of dry matter degraded. Bars represent the SEM of three independent experiments. (B) Quantification of the *rrs* gene copies of the total rumen bacterial population, *F*. *succinogenes* and *R*. *flavefaciens* in DAISY II vessels. Total DNA template extracted from the Daisy II vessel containing only RF and buffer was used as control (Ctrl). The bacterial populations were quantified before and after 24 hours of incubation. pH was monitored at the start and end of incubation. Bars represent the SEM of three independent experiments. Asterisks indicate statistical significance (*: P<0.05; ***: P<0.001).

A potential inhibitory effect of *L*. *reuteri* on the feedstuff degradation capacity of the rumen microbiota was investigated. After 24 hours of incubation, 55% and 43% of corn silage and alfalfa hay dry matter (DM) respectively, were degraded by the rumen microbiota (Fig 4A). Rumen microbiota activity was confirmed by the production of SCFAs after 24 hours of incubation (S4 Fig). Inoculation of *L*. *reuteri* LB1-7 and glycerol supplementation (80 mM) resulted in a significant decrease of DM degradation: 50.4% and 35.8% of corn silage and alfalfa hay DM were degraded, respectively (P<0.05)(Fig 4A), corresponding to inhibition decrease of 8.3 and 16.7%. A shift in SCFA profile was also measured (S4 Fig), indicating a disruption in the rumen microbiota. All incubation vessels had the same initial pH (7.10). After 24 hours of incubation, the pH was slightly decreased (6.70) in the vessels inoculated with *L*. *reuteri* compared with non-inoculated ones (pH 7.05). It is of interest to note that pH 6.70 was still a physiological value for rumen microbiota activity. The decrease in pH value and the fact that lactate and 1,3-PD were recovered in the vessels supplemented with glycerol after 24 hours of LB1-7 incubation (5.2 mM and 7.6 mM, for lactate and 1,3-PD respectively) (S4 Fig) suggested that *L*. *reuteri* remained metabolically active during the course of fermentation.

In view of these results, a potential antimicrobial activity of *L*. *reuteri* against the total rumen bacterial population and two major rumen cellulolytic bacteria, *Fibrobacter succinogenes* and *Ruminococcus flavefaciens*, was investigated (Fig 4B). After 24 hours of incubation, the total bacterial population was slightly greater (P<0.05) with *L*. *reuteri* inoculation compared with control. *R*. *flavefaciens* population decreased during incubation (P<0.05) both in control vessels and in vessels inoculated with *L*. *reuteri* and supplemented with glycerol (decrease of 1 and 0.8 log_10_ *rss* copy per μg of DNA, respectively) (Fig 4B). The *F*. *succinogenes* population remained unchanged in the control vessels during the course of the fermentation whereas a significant decrease (P<0.001) was observed in the vessels inoculated with *L*. *reuteri*. This suggested that *F*. *succinogenes* was more affected by the presence of *L*. *reuteri* than *R*. *flavefaciens*, with on average a decrease of 0.5 log_10_ *rrs* copy per μg of DNA compared to control vessels.

### Survival of EHEC in rectum contents after passage in RF-Glyc80 inoculated with *L*. *reuteri*

**Fig 5:**
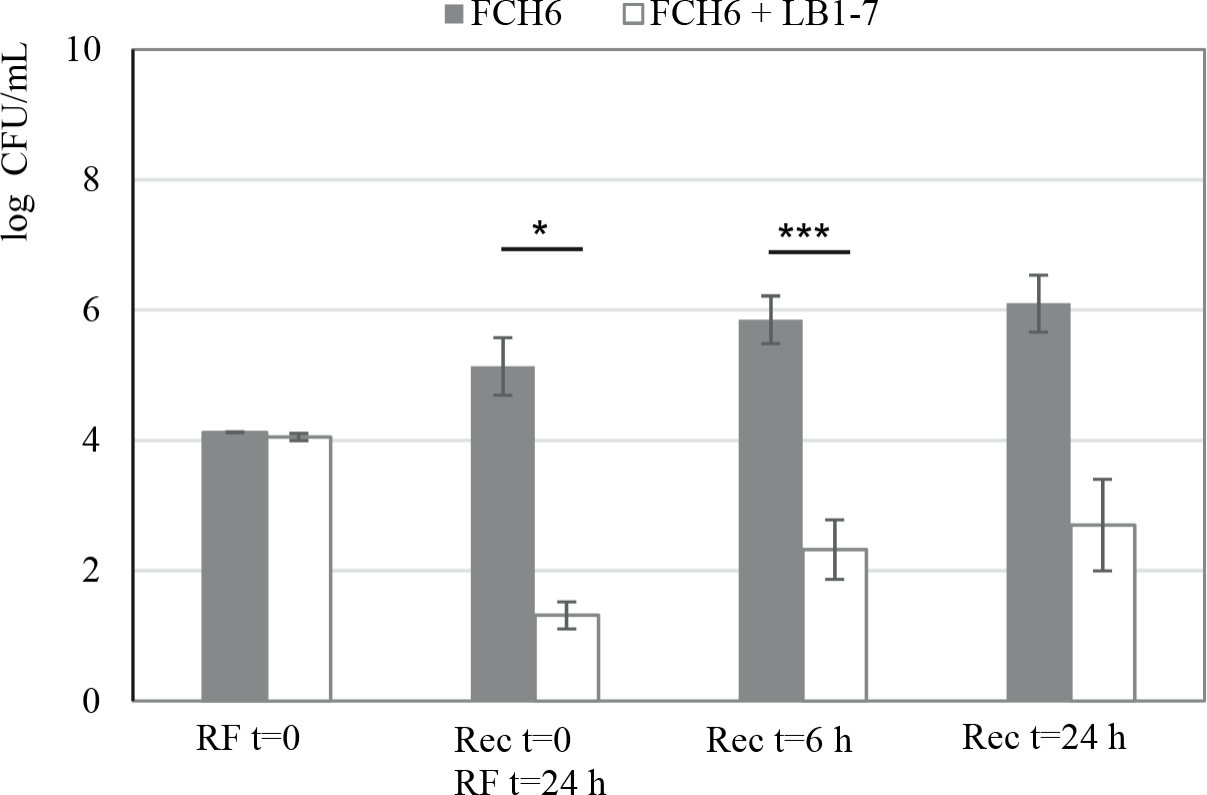
Growth or survival of EHEC in rectum contents after incubation in RF. The strain FCH6 Rif^R^ was first incubated in filter-sterilized RF (FS-RF) samples alone or inoculated with ≈ 10^7^ CFU/mL of *L*. *reuteri* LB1-7 supplemented with 80 mM glycerol under anaerobiosis for 24 hours. The bacterial pellet was then inoculated into Rec samples and incubated under anaerobiosis. RF = 0 represents inoculation of EHEC in FS-RF samples; Rec t = 0 corresponds to RF t = 24h i.e. number of EHEC surviving the incubation in FS-RF during 24 hours; Rec t = 6h and Rec t = 24h correspond to EHEC survival in Rec samples after 6 and 24 hours of incubation respectively. Bars represent the SEM of three independent experiments. Effect of *L*. *reuteri* + Glyc80 is significant *, P<0.05; ***, P<0.001.

Experiments were performed to mimic the passage of EHEC through different segments of the bovine GIT. To this aim, a first incubation of EHEC was performed in filter-sterilized RF (FS-RF) samples followed by a second incubation in Rec samples. The fate of FCH6 Rif^R^ was evaluated after incubation in FS-RF-Glyc80 with *L*. *reuteri* LB1-7 and in following Rec samples. The EHEC strain was also incubated in FS-RF without *L*. *reuteri* and glycerol as a control. A bacterial concentration of 1.3 log_10_ FCH6 Rif^R^ CFU/mL was recovered after 24 hours of incubation in FS-RF-Glyc80 samples inoculated with *L*. *reuteri* LB1-7 (Rec t = 0/RF t = 24h, Fig 5). This concentration was markedly lower than that measured in control RF samples (5.1 log_10_ CFU/mL, P < 0.05) (Rec t = 0/RF t = 24h, Fig 5). Note that, in contrast to incubation in RF samples containing its endogenous microbiota (Figs 1 and 2), *L*. *reuteri* LB1-7 strongly repressed but not totally suppressed the growth of EHEC in FS-RF-Glyc80, suggesting that absence of nutritional competition with the ruminal endogenous microbiota is responsible for the better survival of EHEC. In a second step, bacteria that survived passage through the rumen were inoculated in Rec samples containing its endogenous microbiota. Without prior exposure to *L*. *reuteri* and glycerol in RF, EHEC concentration in Rec samples reached 5.9 and 6.1 log_10_ CFU/mL after 6 and 24 hours of incubation respectively (Fig 5). EHEC concentrations were much lower (2.3 and 2.7 log_10_ CFU/mL, P < 0.001 and P <0.10 respectively) after 6 and 24 hours of incubation in Rec samples following preliminary exposure to LB1-7 in FS-RF-Glyc80 (Fig 5). The results demonstrated that the passage of EHEC in RF in the presence of *L*. *reuteri* and glycerol was efficient to drastically reduce *in vitro* EHEC survival in rectum content.

## Discussion

Strategies can be proposed to reduce the carriage of *E*. *coli* O157:H7 in cattle i) during the animal growth period in order to limit pathogen shedding and thus environmental burden and ii) just before slaughter to limit the contamination of carcasses and thus the burden of *E*. *coli* O157:H7 entering the processing plant [30]. Many studies have tested the use of direct fed microbials (DFMs) as a pre-harvest strategy to reduce fecal shedding of *E*. *coli* O157:H7 in beef cattle [31]. Although results of field studies were variable, a recent meta-analysis showed that distribution of lactic acid bacteria appeared efficient in reducing the prevalence of *E*. *coli* O157:H7 fecal shedding [30].

To our knowledge, the use of *L*. *reuteri* to reduce EHEC survival in bovine gastrointestinal fluids is investigated for the first time. We have compared several *L*. *reuteri* strains and selected *L*. *reuteri* LB1-7 able to convert glycerol to HPA and to suppress EHEC in RF containing metabolically active endogenous microbiota. *L*. *reuteri* LB1-7 appeared to be of particular interest because of its resistance to simulated gastric and intestinal conditions [17], in addition, it can be potentially endowed with probiotic activities as already demonstrated for other lactobacilli isolated from raw milk [32]. Furthermore, in *in vitro* simulation of EHEC fate along the bovine digestive tract, we showed that when the EHEC strain are exposed to *L*. *reuteri* and glycerol in rumen fluid, its viable load sharply decreased, affecting also EHEC viability in a posterior digestive compartment (*i*.*e*. the rectum).

The rumen environment should be appropriate for HPA production by *L*. *reuteri* because it is strictly anaerobic and the concentration of available sugars has been reported to be generally low (<1–2 mM post feeding [33]), a required condition for anaerobic glycerol fermentation by *L*. *reuteri* (a low glucose concentration favours HPA instead of 1,3-PD accumulation [34]). Indeed, the soluble carbohydrates resulting from polysaccharide breakdown by rumen microbiota are either readily fermented or very rapidly sequestered under the form of oligosaccharides into the cells, preventing soluble sugars to be released in RF [35]. The rumen microbiota is able to produce low levels of 1,3-PD in RF-Glyc80 suggesting transient HPA accumulation. This indicates that some bacteria of the rumen microbiota possess the metabolic pathways to convert glycerol to 1,3-PD via HPA reduction. Among 1,3-PD-producers, *Clostridium butyricum* and *C*. *perfringens* are the most commonly reported mammal intestinal species [36]. Although 1,3-PD production is not described in the rumen of cattle, we speculate that members of the *Clostridium* genus, which belong to the core ruminal bacterial community [37], could be responsible for HPA production in RF-Glyc80. However, RF-Glyc80 alone was not effective in preventing EHEC multiplication suggesting that transient HPA produced by the endogenous microbiota was insufficient to achieve an antimicrobial effect.

As expected, the acetate:propionate ratio decreased in RF-Glyc80 after 24 hours of incubation indicating that part of the glycerol was fermented by the rumen microbiota. Several ruminal bacterial species such as *Anaerovibrio lipolytica* and *Selenomonas ruminantium* are able to ferment glycerol into propionate [38–40]. However, incubation of *L*. *reuteri* in RF-Glyc80 resulted in decrease in propionate concentration suggesting that part of the glycerol that could be fermented to propionate by the ruminal microbiota was more efficiently metabolized by *L*. *reuteri*. It can also be hypothesized that the HPA produced by *L*. *reuteri* in RF-Glyc80 resulted in an inhibition of the propionate producing population.

One of the main functions of the rumen microbiota is the degradation and fermentation of plant biomass into short chain fatty acids providing energy for the animal [41]. Therefore, it is important to analyze the effects of LB1-7 inoculation on the degrading activity of the rumen microbiota. In our study, a significant decrease in corn silage and alfalfa hay degradation in the presence of *L*. *reuteri* LB1-7 and glycerol was observed. This decrease is probably due to an inhibitory effect of HPA on the rumen fibrolytic populations, as observed for *F*. *succinogenes*. However, qPCR assay targeting bacterial DNA, as well as DM degradation quantification could lead to underestimation of the effect of *L*. *reuteri* on the rumen bacteria, due to contribution of DNA and active enzymes released by dead bacterial cells. Fermentation end-products profiles also suggested that *L*. *reuteri* exerted an antimicrobial activity against the rumen endogenous microbiota in the presence of glycerol. In addition to the effect on fibrolytic populations, production of HPA by *L*. *reuteri* may lead to inhibition of lactate-utilizing and butyrate- and propionate-producing rumen bacteria, resulting in perturbed fermentations. Consequently, a potential supplementation of *L*. *reuteri* and glycerol during the animal growing period appears to be unsuitable. However, since EHEC carriage at the time of slaughter represents the potential entry of the pathogen into the meat production process, administration of *L*. *reuteri* and glycerol could be considered in view of application to finishing beef cattle, a few days before slaughter, when performance goals (decreased rate of gain—finished weight) are achieved. However, the pre-slaughter glycerol and *L*. *reuteri* administration should avoid additional stressing factors that may have a negative impact on meat quality [42]. Therefore, the approach we suggest for reducing *E*. *coli* shedding by cattle needs to be carefully evaluated to assess the stress responses in living animal and in post-mortem muscle metabolism, in addition to killing activity against EHEC.

Accumulation of lactate was observed in RF-Glyc80 after incubation of *L*. *reuteri* under anaerobiosis. The efficiency of lactate in inhibiting *E*. *coli* O157:H7 multiplication in food products and cattle hides is well documented [43, 44]. Furthermore, Ogawa *et al*. showed that the bactericidal activity of *L*. *casei* against *E*. *coli* O157:H7 was due to production of undissociated lactate, which permeates the bacterial membrane by diffusion and releases protons into the cell [26]. Our data clearly showed that lactate was not involved in EHEC suppression in rumen contents, probably because the weak level of the corresponding undissociated form was below the minimal inhibitory concentration [26]. Nonetheless, it is possible that the distribution of glycerol and *L*. *reuteri* to ruminants leads to EHEC inhibition due to HPA as well as lactate production in the rumen. In further steps, it will be important to analyze the impact of *L*. *reuteri* and glycerol association *in vivo*, in order to assess their effects on bovine gastrointestinal health due to lactate production and microbiota disruption.

The potential use of *L*. *reuteri* as a feed supplement to reduce EHEC burden in cattle would necessarily be associated with glycerol administration. It is generally admitted that supplementing feed with a formulation containing freeze-dried micro-organisms is easy to implement, as far as strains stability issues are resolved. Introduction of *L*. *reuteri* as direct-fed microbial (DFM) would be of interest because the use of DFMs is widely accepted in ruminant nutrition and is perceived as a natural non-antibiotic way of improving animal performance and health [45]. The use of glycerol as cattle feed supplement has already been proposed to improve animal performance because of its cost-effective energetic value. Because the energy content of glycerol is close to that of corn, the replacement of corn by dry glycerol in cows daily supplementation has been considered [46]. Furthermore, the glycerol concentrations needed for EHEC inhibition by *L*. *reuteri in vitro* are close to those used in cattle production [46, 47]. However, *in vivo* experiments will be needed to determine the glycerol concentration required for production of sufficient HPA to suppress EHEC in the rumen of cows, as part of the glycerol might be absorbed across the rumen wall [48].

In conclusion, *L*. *reuteri* LB1-7 appeared to resist the physical and microbiological conditions encountered in the rumen of cows and was effective in suppressing EHEC O157:H7 in RF *in vitro*. Data presented in this report define valuable information on the optimal antagonistic effect of a selected *L*. *reuteri* strain against EHEC (*L*. *reuteri*-EHEC concentration ratio, glycerol level, aeration conditions) and provide helpful data to set up more targeted *in vivo* trials to assess the efficiency of *L*. *reuteri* to decrease EHEC shedding by cattle. Future studies will be required to determine i) DFM supplementation parameters, ii) the effect of *L*. *reuteri* and glycerol on rumen microbiota and iii) the survival of EHEC strains in lower gastrointestinal tract. *In vivo* studies will also be necessary to explore the potential of HPA-producing *L*. *reuteri* alone or in combination with already commercialized DFMs in order to ultimately validate an efficient pre-harvest strategy in beef cattle with the aim to improve food safety.

## Supporting information

S1 Fig Survival of EHEC strains in RF samples. The strains FCH6 Rif^R^ and EDL933 Rif^R^ were incubated in RF samples for 24 hours under anaerobiosis before enumeration. Bars represent the SEM of three independent experiments. Asterisks indicate statistical significance (*: *P<0*.05; ***: *P<0*.*001)*.
(TIF)

S2 Fig Agar spot assays of *L*. *reuteri* strains. *L*. *reuteri* strains (LB1-7, F275, 65-A, F70 and 100–23) were first spotted onto the surface of Brain Heart Infusion (BHI) agar supplemented with 20 mM glucose and incubated anaerobically. The EHEC strain FCH6 was then inoculated in soft agar with or without glycerol (2%) and poured over the *L*. *reuteri* spots (in order to facilitate or not HPA production by spots containing *L*. *reuteri*). The plates were then incubated and the antimicrobial activity was recorded as growth-free inhibition zones around the spots as previously described [20].
(TIF)

S3 Fig Amplification of DNA extracted from *L*. *reuteri* strains. PCR detection of the genes *gldC* (A) and *dhaT* (B) from genomic DNA extracted from *L*. *reuteri* strains. Lane 1, molecular size marker (MassRuler DNA Ladder, ThermoFisher Scientific); lane 2, *L*. *reuteri* LB1-7; lane 3, *L*. *reuteri* 65A; lane 4, *L*. *reuteri* F70; lane 5, *L*. *reuteri* F275; line 6, *L*. *reuteri* 100–23.
The PCR products were subjected to electrophoresis on 1% agarose gel and visualized by ethidium bromide staining.
(TIF)

S4 Fig Fermentation end-products in Daisy II experiments. The Daisy II method was used to analyze the degrading activity of the rumen microbiota. The Daisy II vessels containing RF were supplemented or not with 80 mM glycerol and inoculated or not with *L*. *reuteri* LB1-7. The fermentation end-products were quantified in Daisy II vessels before and after 24 hours of anaerobic incubation. Bars represent the SEM of three independent experiments. Asterisks indicate statistical significance (*: P<0.05; ***: P<0.001).
ACE: acetate; PROP: propionate; BUTY: butyrate; GLY: glycerol; LACT: lactate.
(TIF)

S5 Fig Growth or survival of EHEC in samples collected from the rectum of cows. The strain FCH6 Rif^*R*^ was incubated under anaerobiosis in Rec samples or co-incubated in Rec samples inoculated with *L*. *reuteri* LB1-7 (≈ 10^*7*^ CFU/mL) and supplemented with 80 mM glycerol. Bars represent the SEM of three independent experiments. Effect of *L*. *reuteri* + Glyc80 is significant *, P<0.05.
(TIF)

S1 Table Bacterial strains used in this study. ^*a*^: DSM, Deutsche Sammlung von Mikroorganismen und Zellkulturen, Braunschweig, Germany.
^*b*^: ISPA, Institute of Sciences of Food Production microbial collection number; National Research Council, Italy.
(DOCX)

S2 Table Oligonucleotides used in this study. DNA sequences from *L*. *reuteri* available in DNA databases (JCM 1112 [AP007282.1], DSM 20016 [CP000705.1], FUA3400 [KJ435307.1], SD2112 [CP002844.1], BR11 [GU191838.1] and BPL36 [JQ897939.1]) were aligned (Align Sequences Nucleotide BLAST [*http*:*//blast*.*ncbi*.*nlm*.*nih*.*gov*]) to design the primer pairs used to amplify *gldC* and *dhaT*.
(DOCX)

S3 Table EHEC inhibition in the presence of *L*. *reuteri* and glycerol. (DOCX)
