# Supplementary figures and images for: Lactobacillus reuteri suppresses E. coli O157:H7 in bovine ruminal fluid: Toward a pre-slaughter strategy to improve food safety?
Source: PLoS One. 2017 Nov 1;12(11):e0187229. doi: 10.1371/journal.pone.0187229 (PMC5665532; doi:10.1371/journal.pone.0187229)

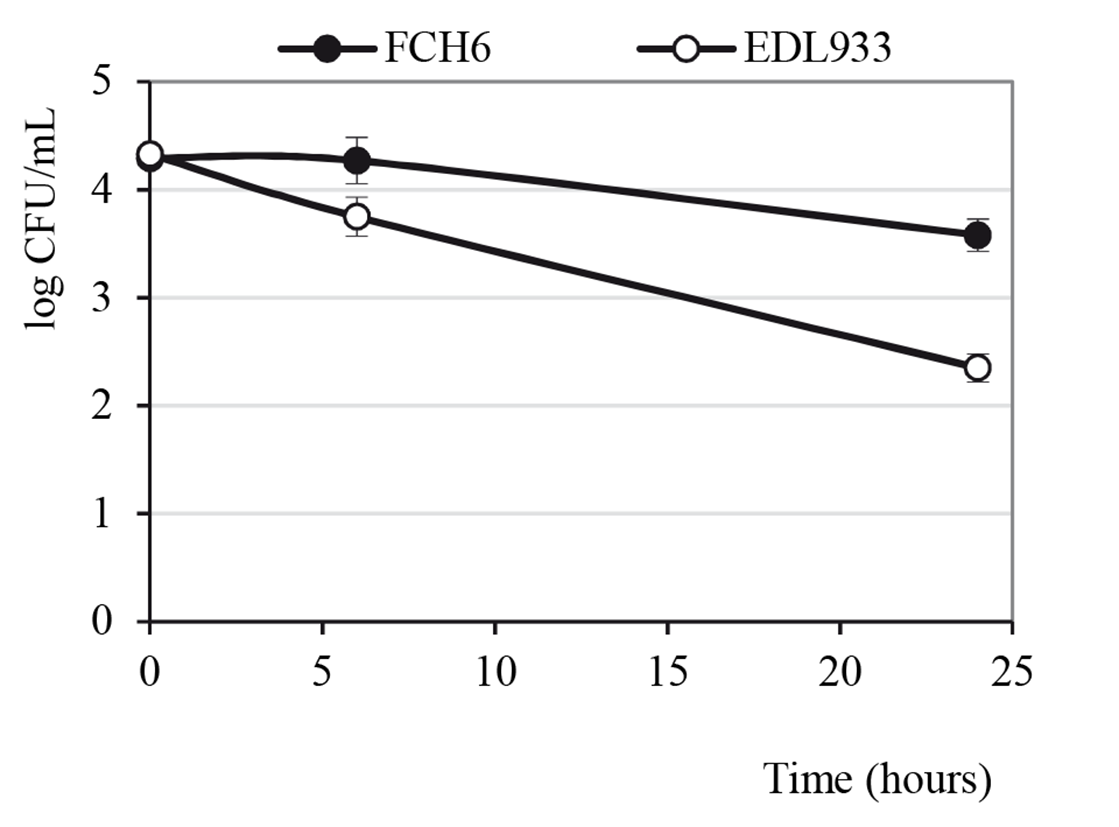

Supplement: S1 Fig — The strains FCH6 RifR and EDL933 RifR were incubated in RF samples for 24 hours under anaerobiosis before enumeration. Bars represent the SEM of three independent experiments. Asterisks indicate statistical significance (*: P<0.05; ***: P<0.001). (TIF) [file pone.0187229.s001.tif]

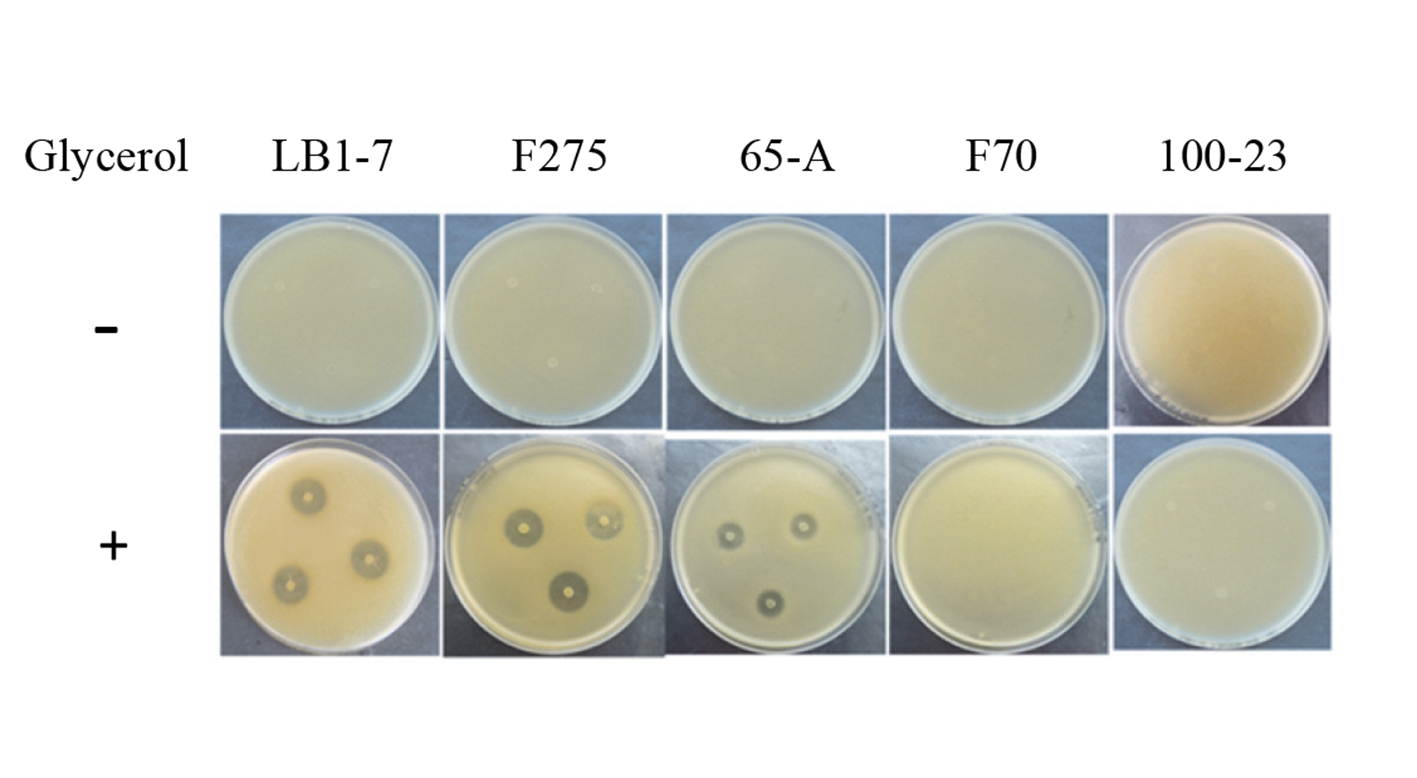

Supplement: S2 Fig — L. reuteri strains (LB1-7, F275, 65-A, F70 and 100–23) were first spotted onto the surface of Brain Heart Infusion (BHI) agar supplemented with 20 mM glucose and incubated anaerobically. The EHEC strain FCH6 was then inoculated in soft agar with or without glycerol (2%) and poured over the L. reuteri spots (in order to facilitate or not HPA production by spots containing L. reuteri). The plates were then incubated and the antimicrobial activity was recorded as growth-free inhibition zones around the spots as previously described [20]. (TIF) [file pone.0187229.s002.tif]

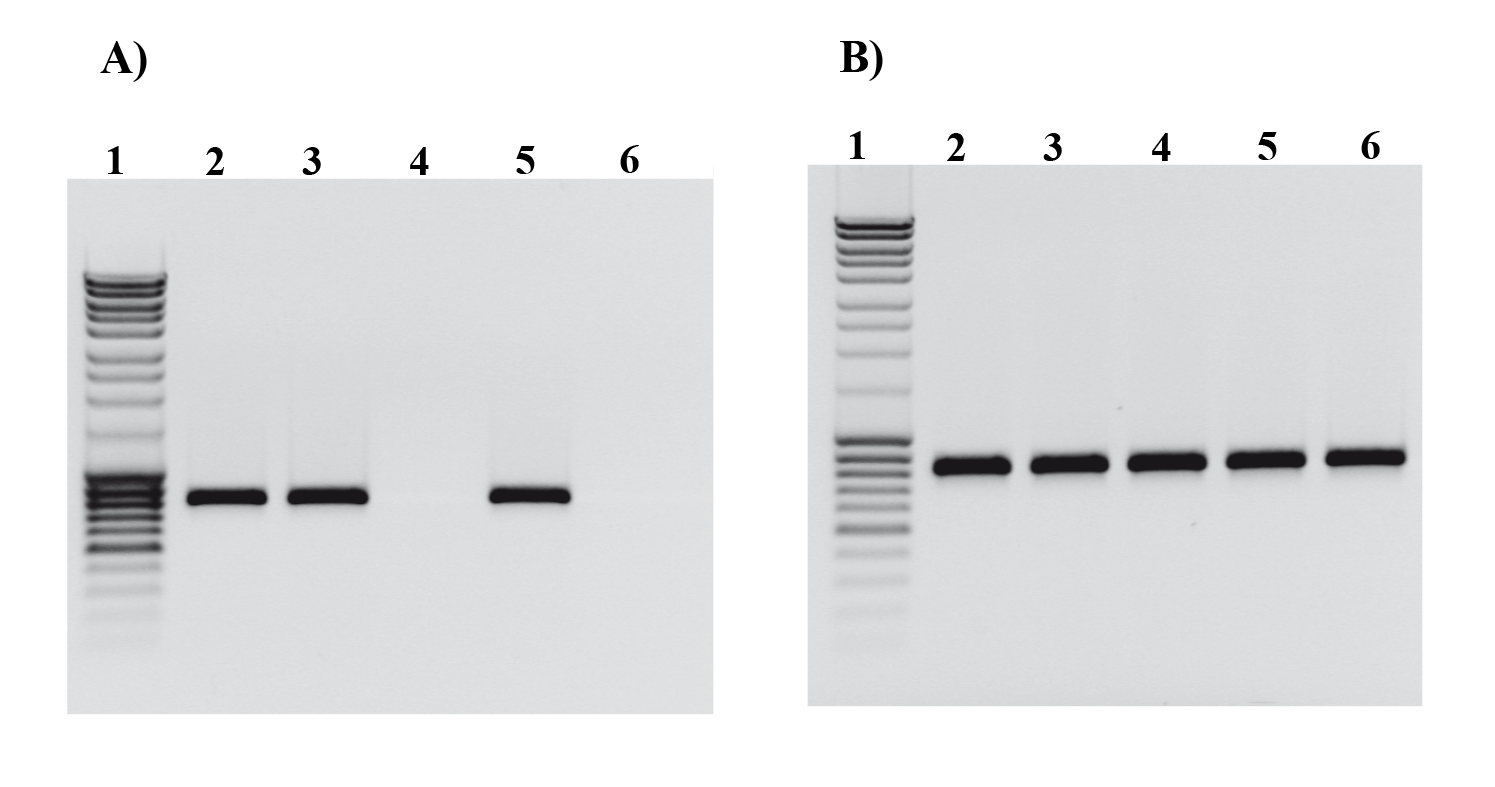

Supplement: S3 Fig — PCR detection of the genes gldC (A) and dhaT (B) from genomic DNA extracted from L. reuteri strains. Lane 1, molecular size marker (MassRuler DNA Ladder, ThermoFisher Scientific); lane 2, L. reuteri LB1-7; lane 3, L. reuteri 65A; lane 4, L. reuteri F70; lane 5, L. reuteri F275; line 6, L. reuteri 100–23. The PCR products were subjected to electrophoresis on 1% agarose gel and visualized by ethidium bromide staining. (TIF) [file pone.0187229.s003.tif]

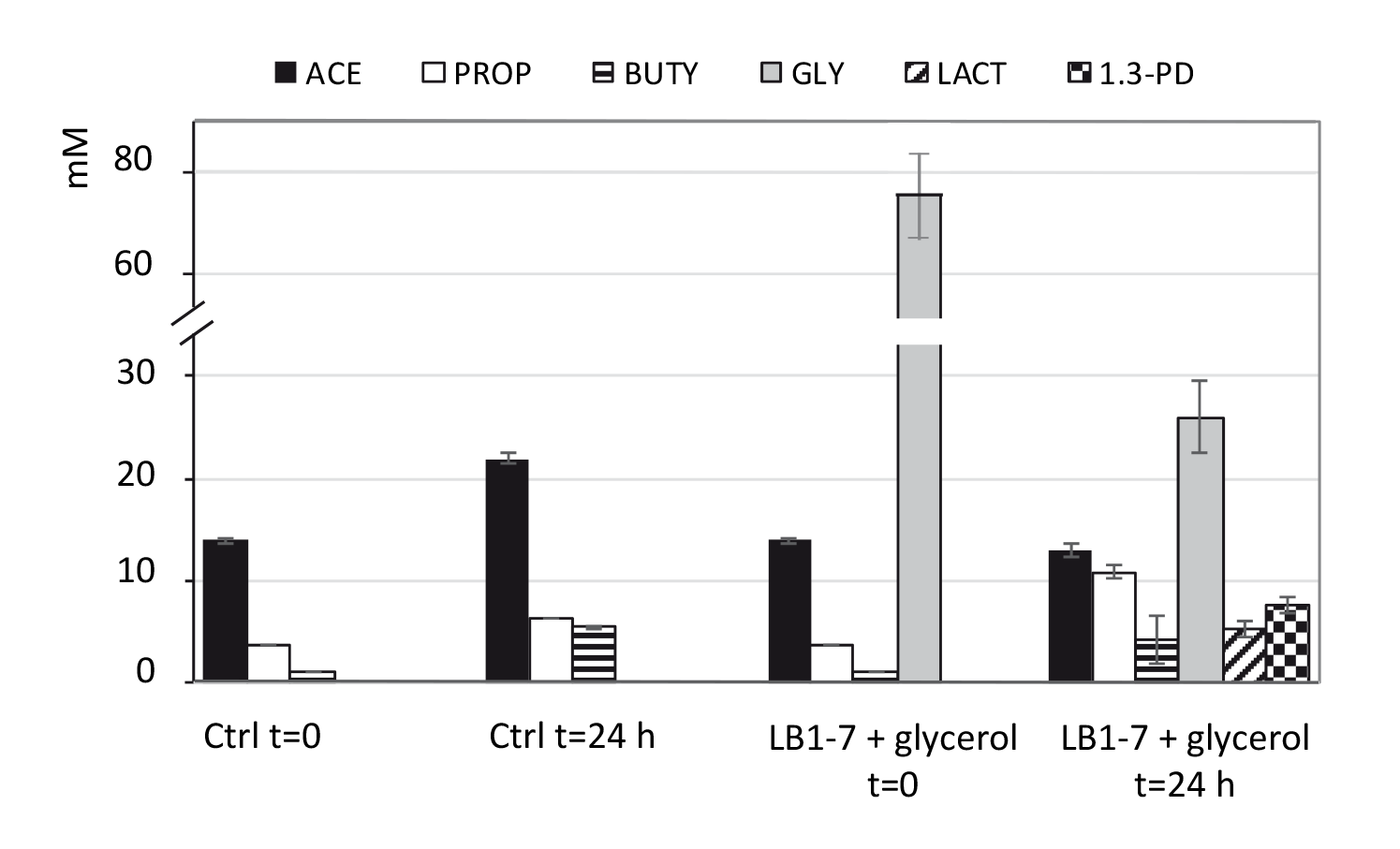

Supplement: S4 Fig — The Daisy II method was used to analyze the degrading activity of the rumen microbiota. The Daisy II vessels containing RF were supplemented or not with 80 mM glycerol and inoculated or not with L. reuteri LB1-7. The fermentation end-products were quantified in Daisy II vessels before and after 24 hours of anaerobic incubation. Bars represent the SEM of three independent experiments. Asterisks indicate statistical significance (*: P<0.05; ***: P<0.001). ACE: acetate; PROP: propionate; BUTY: butyrate; GLY: glycerol; LACT: lactate. (TIF) [file pone.0187229.s004.tif]

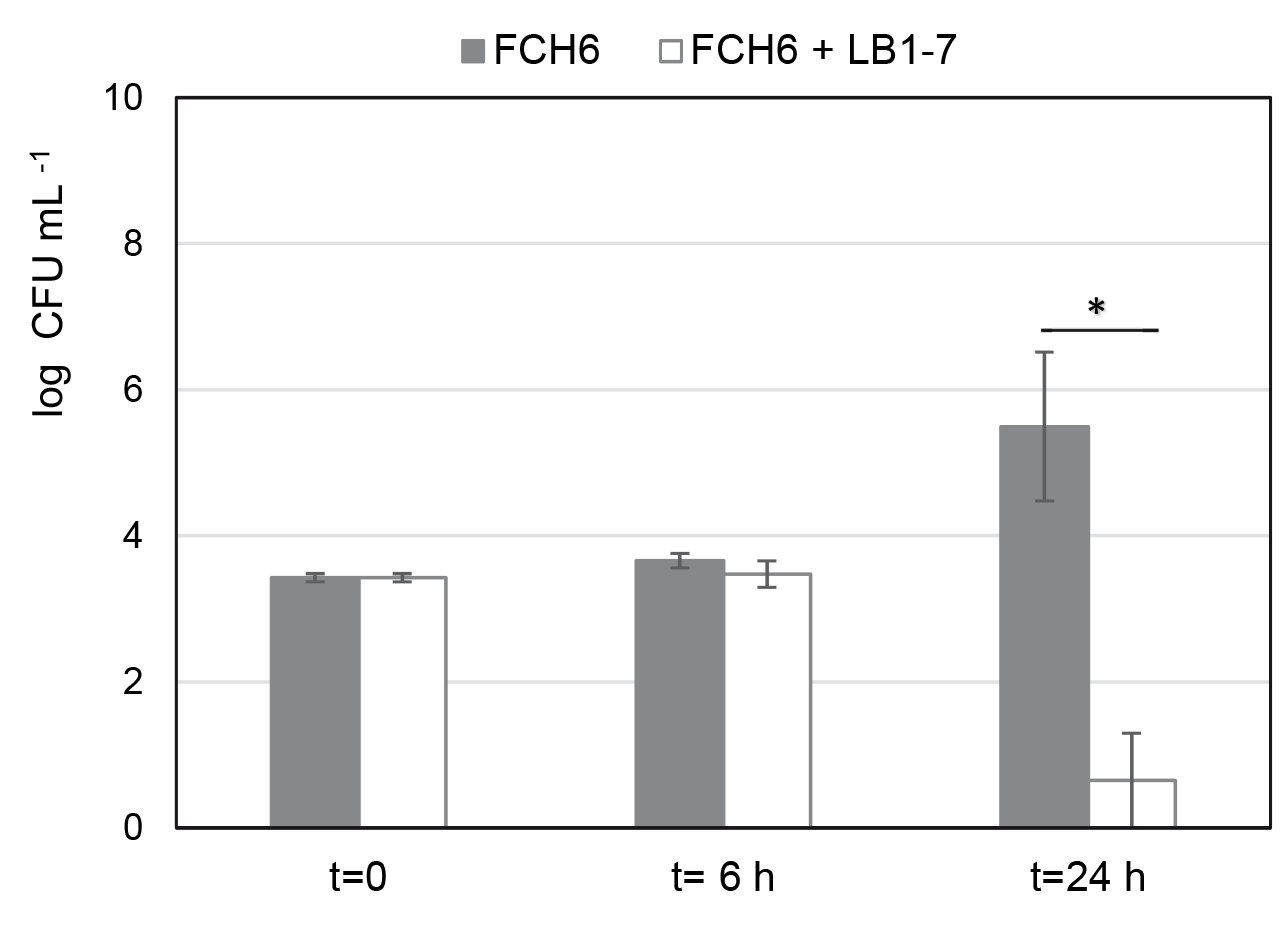

Supplement: S5 Fig — The strain FCH6 RifR was incubated under anaerobiosis in Rec samples or co-incubated in Rec samples inoculated with L. reuteri LB1-7 (≈ 107 CFU/mL) and supplemented with 80 mM glycerol. Bars represent the SEM of three independent experiments. Effect of L. reuteri + Glyc80 is significant *, P<0.05. (TIF) [file pone.0187229.s005.tif]
