# Supplementary material for: Lactobacillus reuteri suppresses E. coli O157:H7 in bovine ruminal fluid: Toward a pre-slaughter strategy to improve food safety?
Source: PLoS One. 2017 Nov 1;12(11):e0187229. doi: 10.1371/journal.pone.0187229 (PMC5665532; doi:10.1371/journal.pone.0187229)
Supplement: S1 Table — a: DSM, Deutsche Sammlung von Mikroorganismen und Zellkulturen, Braunschweig, Germany. b: ISPA, Institute of Sciences of Food Production microbial collection number; National Research Council, Italy. (DOCX) [file pone.0187229.s006.docx]

**S1 Table Bacterial strains used in this study.**

| **Strains** | **Description** | **Source or reference** |
| --- | --- | --- |
| ***L. reuteri strains*** |  |  |
| 100-23 | *L. reuteri* isolated from the digestive tract of a rat | DSM 17509 ^a^ |
| 65A | *L. reuteri* isolated from rat faeces | DSM 20056 ^a^ |
| F70 | *L. reuteri* isolated from human faeces | DSM 20053 ^a^ |
| F275 | *L. reuteri* isolated from human faeces | DSM 20016 ^a^ |
| LB1-7 | *L. reuteri* isolated from raw bovine milk | ISPA 13519 ^b^ |
| LB1-7 Rif^R^ | Spontaneous rifampicin resistant mutant of LB1-7 | This study |
|  |  |  |
| **EHEC strains** |  |  |
| FCH6 | EHEC O157:H7 isolated from milk goat cheese | This study |
| EDL933 | EHEC O157:H7 isolated from raw hamburger meat | Riley *et al.,* 1983 |
| FCH6 Rif^R^ | Spontaneous rifampicin resistant mutant of FCH6 | This study |
| EDL933 Rif^R^ | Spontaneous rifampicin resistant mutant of EDL933 | Chaucheyras-Durand *et al.,* 2006 |

Chaucheyras-Durand F, Madic J, Doudin F, Martin C. Biotic and abiotic factors influencing *in vitro* growth of *Escherichia coli* O157:H7 in ruminant digestive contents. Applied and Environmental Microbiology. 2006; 72:4136–4142.

Riley LW, [Remis RS](http://www.ncbi.nlm.nih.gov/pubmed/?term=Remis%20RS%5BAuthor%5D&cauthor=true&cauthor_uid=6338386), [Helgerson SD](http://www.ncbi.nlm.nih.gov/pubmed/?term=Helgerson%20SD%5BAuthor%5D&cauthor=true&cauthor_uid=6338386), [McGee HB](http://www.ncbi.nlm.nih.gov/pubmed/?term=McGee%20HB%5BAuthor%5D&cauthor=true&cauthor_uid=6338386), [Wells JG](http://www.ncbi.nlm.nih.gov/pubmed/?term=Wells%20JG%5BAuthor%5D&cauthor=true&cauthor_uid=6338386), [Davis BR](http://www.ncbi.nlm.nih.gov/pubmed/?term=Davis%20BR%5BAuthor%5D&cauthor=true&cauthor_uid=6338386), et al. [Hemorrhagic colitis associated with a rare *Escherichia coli* serotype.](http://www.ncbi.nlm.nih.gov/pubmed/6338386) New England Journal of Medicine. 1983; 308:681-685.
