# Supplementary material for: Lactobacillus reuteri suppresses E. coli O157:H7 in bovine ruminal fluid: Toward a pre-slaughter strategy to improve food safety?
Source: PLoS One. 2017 Nov 1;12(11):e0187229. doi: 10.1371/journal.pone.0187229 (PMC5665532; doi:10.1371/journal.pone.0187229)
Supplement: S2 Table — DNA sequences from L. reuteri available in DNA databases (JCM 1112 [AP007282.1], DSM 20016 [CP000705.1], FUA3400 [KJ435307.1], SD2112 [CP002844.1], BR11 [GU191838.1] and BPL36 [JQ897939.1]) were aligned (Align Sequences Nucleotide BLAST [http://blast.ncbi.nlm.nih.gov]) to design the primer pairs used to amplify gldC and dhaT. (DOCX) [file pone.0187229.s007.docx]

**S2 Table Oligonucleotides used in this study.**

|  | **Sequence 5’-3’** | **Amplicon size (bp)** | **Target** | **Reference** |
| --- | --- | --- | --- | --- |
| **Bacterial quantification** |  |  |  |  |
|  |  |  |  |  |
| 520F | AGCAGCCGCGGTAAT | 280 | Global population | Edwards *et al.,* 2007 |
| 799R2cor | CAGGGTATCTAATCCTGTT |  |  |  |
|  |  |  |  |  |
| Lab 159 | GGAAACAGATGCTAATACCG | 518 | *Lactobacillus* group | Heilig *et al.,* 2002 |
| Lab 677 | CACCGCTACACATGGAG |  |  |  |
|  |  |  |  |  |
| HspFORReuteri | GCGTTGATGTTGTTGAAGGAATGAGCTTTG | 200 | *L. reuteri* | Herbel *et al.,* 2013 |
| HspREVReuteri | CATCAGCAATGATTAAGAGAGCACGGCC |  |  |  |
|  |  |  |  |  |
| Fs2006F | GTTCGGAATTACTGGGCGTAAA | 121 | *F. succinogenes* | Denman *et al.,* 2006 |
| Fs2006R | CGCCTGCCCCTGAACTATC |  |  |  |
|  |  |  |  |  |
| Rf2006F | CGAACGGAGATAATTTGAGTTTACTTAGG | 132 | *R. flavefaciens* | Denman *et al.,* 2006 |
| Rf2006R | CGGTCTCTGTATGTTATGAGGTATTACC |  |  |  |
|  |  |  |  |  |
| **Gene detection** |  |  |  |  |
| gldC-F | GTTCTGTTGAAGAAGCTACTG | 795 | Glycerol dehydratase | This study |
| gldC-R | GATCCATCATATCTTGAGCAG |  |  |  |
|  |  |  |  |  |
| dhaT-F | CAATGGTCCTGTAAAGCAAAC | 850 | Propanediol dehydrogenase | This study |
| dhaT-R | GCTTCGTAAAGGGTAAGACC |  |  |  |
|  |  |  |  |  |
| VT1-F | CATTGTCTGGTGACAGTAGCT | 732 | Stx1 toxin | Gannon *et al.,* 1997 |
| VT1-R | CCCGTAATTTGCGCACTGAG |  |  |  |
|  |  |  |  |  |
| LP43 | ATCCTATTCCCGGGAGTTTACG | 587 | Stx2 toxin | Cebula et al., 1995 |
| LP44 | GCGTCATCGTATACACAGGAGC |  |  |  |
|  |  |  |  |  |
| FLICH7-F | GCGCTGTCGAGTTCTATCGAGC | 625 | Flagellar H7 antigen | Gannon *et al.,* 1997 |
| FLICH7-R | CAACGGTGACTTTATCGCCATTCC |  |  |  |
|  |  |  |  |  |
| RfbE-O157-F | AAGATTGCGCTGAAGCCTTTG | 497 | O157 antigen | Desmarchelier *et al.,* 1998 |
| RfbE-O157-R | CATTGGCATCGTGTGGACAG |  |  |  |

[Cebula TA](http://www.ncbi.nlm.nih.gov/pubmed/?term=Cebula%20TA%5BAuthor%5D&cauthor=true&cauthor_uid=7535315), [Payne WL](http://www.ncbi.nlm.nih.gov/pubmed/?term=Payne%20WL%5BAuthor%5D&cauthor=true&cauthor_uid=7535315), [Feng P](http://www.ncbi.nlm.nih.gov/pubmed/?term=Feng%20P%5BAuthor%5D&cauthor=true&cauthor_uid=7535315). Simultaneous identification of strains of *Escherichia coli* serotype O157:H7 and their Shiga-like toxin type by mismatch amplification mutation assay-multiplex PCR. Journal of Clinical Microbiology. 1995; 33:248-250.

Denman SE, McSweeney CS. [Development of a real-time PCR assay for monitoring anaerobic fungal and cellulolytic bacterial populations within the rumen.](http://www.ncbi.nlm.nih.gov/pubmed/17117998) [FEMS Microbiology Ecology. 2006;](http://www.ncbi.nlm.nih.gov/pubmed/17941835) 58:572-582.

Desmarchelier PM, Bilge SS, Fegan N, Mills L, Vary JC Jr., Tarr PI. [A PCR specific for *Escherichia coli* O157 based on the *rfb* locus encoding O157 lipopolysaccharide.](http://www.ncbi.nlm.nih.gov/pubmed/9620428) Journal of Clinical Microbiology. 1998; 36:1801-1804.

[Edwards JE](http://www.ncbi.nlm.nih.gov/pubmed/?term=Edwards%20JE%5BAuthor%5D&cauthor=true&cauthor_uid=17941835), [Huws SA](http://www.ncbi.nlm.nih.gov/pubmed/?term=Huws%20SA%5BAuthor%5D&cauthor=true&cauthor_uid=17941835), [Kim EJ](http://www.ncbi.nlm.nih.gov/pubmed/?term=Kim%20EJ%5BAuthor%5D&cauthor=true&cauthor_uid=17941835), [Kingston-Smith AH](http://www.ncbi.nlm.nih.gov/pubmed/?term=Kingston-Smith%20AH%5BAuthor%5D&cauthor=true&cauthor_uid=17941835). Characterization of the dynamics of initial bacterial colonization of nonconserved forage in the bovine rumen. [FEMS Microbiology Ecology.](http://www.ncbi.nlm.nih.gov/pubmed/17941835) 2007; 62:323-335.

[Gannon VP](http://www.ncbi.nlm.nih.gov/pubmed/?term=Gannon%20VP%5BAuthor%5D&cauthor=true&cauthor_uid=9041407), [D'Souza S](http://www.ncbi.nlm.nih.gov/pubmed/?term=D'Souza%20S%5BAuthor%5D&cauthor=true&cauthor_uid=9041407), [Graham T](http://www.ncbi.nlm.nih.gov/pubmed/?term=Graham%20T%5BAuthor%5D&cauthor=true&cauthor_uid=9041407), [King RK](http://www.ncbi.nlm.nih.gov/pubmed/?term=King%20RK%5BAuthor%5D&cauthor=true&cauthor_uid=9041407), [Rahn K](http://www.ncbi.nlm.nih.gov/pubmed/?term=Rahn%20K%5BAuthor%5D&cauthor=true&cauthor_uid=9041407), [Read S](http://www.ncbi.nlm.nih.gov/pubmed/?term=Read%20S%5BAuthor%5D&cauthor=true&cauthor_uid=9041407). Use of the flagellar H7 gene as a target in multiplex PCR assays and improved specificity in identification of enterohemorrhagic *Escherichia coli* strains. Journal of Clinical Microbiology. 1997; 35:656-662.

[Heilig HG](https://www.ncbi.nlm.nih.gov/pubmed/?term=Heilig%20HG%5BAuthor%5D&cauthor=true&cauthor_uid=11772617), [Zoetendal EG](https://www.ncbi.nlm.nih.gov/pubmed/?term=Zoetendal%20EG%5BAuthor%5D&cauthor=true&cauthor_uid=11772617), [Vaughan EE](https://www.ncbi.nlm.nih.gov/pubmed/?term=Vaughan%20EE%5BAuthor%5D&cauthor=true&cauthor_uid=11772617), [Marteau P](https://www.ncbi.nlm.nih.gov/pubmed/?term=Marteau%20P%5BAuthor%5D&cauthor=true&cauthor_uid=11772617), [Akkermans AD](https://www.ncbi.nlm.nih.gov/pubmed/?term=Akkermans%20AD%5BAuthor%5D&cauthor=true&cauthor_uid=11772617), [de Vos WM](https://www.ncbi.nlm.nih.gov/pubmed/?term=de%20Vos%20WM%5BAuthor%5D&cauthor=true&cauthor_uid=11772617). Molecular diversity of Lactobacillus spp. and other lactic acid bacteria in the human intestine as determined by specific amplification of 16S ribosomal DNA. [Appl Environ Microbiol.](https://www.ncbi.nlm.nih.gov/pubmed/?term=Heilig%2C+2002+lactobacillus) 2002. 68:114-23.

[Herbel SR](http://www.ncbi.nlm.nih.gov/pubmed/?term=Herbel%20SR%5BAuthor%5D&cauthor=true&cauthor_uid=24024971), [Lauzat B](http://www.ncbi.nlm.nih.gov/pubmed/?term=Lauzat%20B%5BAuthor%5D&cauthor=true&cauthor_uid=24024971), [von Nickisch-Rosenegk M](http://www.ncbi.nlm.nih.gov/pubmed/?term=von%20Nickisch-Rosenegk%20M%5BAuthor%5D&cauthor=true&cauthor_uid=24024971), [Kuhn M](http://www.ncbi.nlm.nih.gov/pubmed/?term=Kuhn%20M%5BAuthor%5D&cauthor=true&cauthor_uid=24024971), [Murugaiyan J](http://www.ncbi.nlm.nih.gov/pubmed/?term=Murugaiyan%20J%5BAuthor%5D&cauthor=true&cauthor_uid=24024971), [Wieler LH](http://www.ncbi.nlm.nih.gov/pubmed/?term=Wieler%20LH%5BAuthor%5D&cauthor=true&cauthor_uid=24024971), et al. [Species-specific quantification of probiotic lactobacilli in yoghurt by quantitative real-time PCR.](http://www.ncbi.nlm.nih.gov/pubmed/24024971) Journal of Applied Microbiology. 2013; 115:1402-1410.
