# Supplementary material for: Lactobacillus reuteri suppresses E. coli O157:H7 in bovine ruminal fluid: Toward a pre-slaughter strategy to improve food safety?
Source: PLoS One. 2017 Nov 1;12(11):e0187229. doi: 10.1371/journal.pone.0187229 (PMC5665532; doi:10.1371/journal.pone.0187229)
Supplement: S3 Table — (DOCX) [file pone.0187229.s008.docx]

**S3 Table EHEC inhibition in the presence of *L. reuteri* and glycerol.**

| Glycerol (mM) | *L. reuteri* LB1-7 (bacteria/mL) | FCH6 Rif^R^ (bacteria/mL ) | EHEC inhibition |
| --- | --- | --- | --- |
| 10 | 10 ^5^ | 10 ^4^ | - |
| 20 | 10 ^5^ | 10 ^4^ | - |
| 40 | 10 ^5^ | 10 ^4^ | - |
| 80 | 10 ^5^ | 10 ^4^ | - |
| 160 | 10 ^5^ | 10 ^4^ | - |
| 10 | 10 ^6^ | 10 ^4^ | - |
| 20 | 10 ^6^ | 10 ^4^ | - |
| 40 | 10 ^6^ | 10 ^4^ | - |
| 80 | 10 ^6^ | 10 ^4^ | - |
| 160 | 10 ^6^ | 10 ^4^ | - |
| 10 | 10 ^7^ | 10 ^4^ | - |
| 20 | 10 ^7^ | 10 ^4^ | - |
| 40 | 10 ^7^ | 10 ^4^ | ± |
| 80 | 10 ^7^ | 10 ^4^ | +++ |
| 160 | 10 ^7^ | 10 ^4^ | +++ |
